# Activation of pro-survival metabolic networks by 1,25(OH)_2_D_3_ does not hamper the sensitivity of breast cancer cells to chemotherapeutics

**DOI:** 10.1186/s40170-018-0183-6

**Published:** 2018-08-30

**Authors:** Mohamed A. Abu el Maaty, Yasamin Dabiri, Fadi Almouhanna, Biljana Blagojevic, Jannick Theobald, Michael Büttner, Stefan Wölfl

**Affiliations:** 1 Institute of Pharmacy and Molecular Biotechnology, Heidelberg University, Im Neuenheimer Feld 364, 69120 Heidelberg, Germany; 2 Metabolomics Core Technology Platform, Center for Organismal Studies (COS), Heidelberg University, Im Neuenheimer Feld 360, 69120 Heidelberg, Germany

**Keywords:** Vitamin D, Breast cancer, Metabolism, G6PD, TXNIP, AMPK, Serine, ITCH, Estrogen receptor

## Abstract

**Background:**

We have previously identified 1,25-dihydroxyvitamin D_3_ [1,25(OH)_2_D_3_], the bioactive form of vitamin D_3_, as a potent regulator of energy-utilization and nutrient-sensing pathways in prostate cancer cells. In the current study, we investigated the effects of 1,25(OH)_2_D_3_ on breast cancer (BCa) cell metabolism using cell lines representing distinct molecular subtypes, luminal (MCF-7 and T-47D), and triple-negative BCa (MDA-MB-231, MDA-MB-468, and HCC-1143).

**Methods:**

1,25(OH)_2_D_3_’s effect on BCa cell metabolism was evaluated by employing a combination of real-time measurements of glycolysis/oxygen consumption rates using a biosensor chip system, GC/MS-based metabolomics, gene expression analysis, and assessment of overall energy levels. The influence of treatment on energy-related signaling molecules was investigated by immunoblotting.

**Results:**

We show that 1,25(OH)_2_D_3_ significantly induces the expression and activity of the pentose phosphate pathway enzyme glucose-6-phosphate dehydrogenase (G6PD) in all BCa cell lines, however differentially influences glycolytic and respiratory rates in the same cells. Although 1,25(OH)_2_D_3_ treatment was found to induce seemingly anti-oxidant responses in MCF-7 cells, such as increased intracellular serine levels, and reduce the expression of its putative target gene thioredoxin-interacting protein (TXNIP), intracellular reactive oxygen species levels were found to be elevated. Serine accumulation in 1,25(OH)_2_D_3_-treated cells was not found to hamper the efficacy of chemotherapeutics, including 5-fluorouracil. Detailed analyses of the nature of TXNIP’s regulation by 1,25(OH)_2_D_3_ included genetic and pharmacological inhibition of signaling molecules and metabolic enzymes including AMP-activated protein kinase and G6PD, as well as by studying the ITCH (E3 ubiquitin ligase)-TXNIP interaction. While these investigations demonstrated minimal involvement of such pathways in the observed non-canonical regulation of TXNIP, inhibition of estrogen receptor (ER) signaling by tamoxifen mirrored the reduction of TXNIP levels by 1,25(OH)_2_D_3_, demonstrating that the latter’s negative regulation of ER expression is a potential mechanism of TXNIP modulation.

**Conclusions:**

Altogether, we propose that regulation of energy metabolism contributes to 1,25(OH)_2_D_3_’s anti-cancer effects and that combining 1,25(OH)_2_D_3_ with drugs targeting metabolic networks in tumor cells may lead to synergistic effects.

**Electronic supplementary material:**

The online version of this article (10.1186/s40170-018-0183-6) contains supplementary material, which is available to authorized users.

## Background

Breast cancer (BCa) is the main cause of cancer death in females in both developed and developing countries [1]. Several risk factors have been implicated in the pathogenesis of the disease, including lifestyle-related factors, for example smoking, obesity, and physical inactivity, as well as genetic factors, like mutations in breast cancer susceptibility genes (*BRCA 1* and *2*) [1, 2]. In addition to these risk factors, mounting evidence has demonstrated that vitamin D deficiency contributes to both BCa incidence and survival [3, 4].

Vitamin D is a seco-steroid that is produced in humans upon exposure of the skin to ultraviolet-B radiation [5]. The resulting hormonal precursor molecule is further metabolized to yield its hormonally active form—1,25-dihydroxyvitamin D (1,25(OH)_2_D_3_, also known as calcitriol)—through a hydroxylation step in the liver, followed by a second activating hydroxylation in the kidneys [5]. 1,25(OH)_2_D_3_ subsequently binds to its nuclear vitamin D receptor (VDR), present in diverse cell types, and regulates the expression of hundreds of genes known to influence proliferation, differentiation, and angiogenesis [5]. Both in vitro and animal studies have illustrated that 1,25(OH)_2_D_3_ and its analogues are promising chemotherapeutics against BCa [3, 6], with reports showing induction of cell cycle arrest and apoptosis, as well as regulation of estrogen signaling with treatment [3, 6]. Additionally, studies have shown that VDR expression inversely correlates with breast cancer mortality and that CYP24A1, the vitamin D-catabolizing enzyme, is a putative oncogene [6].

BCa is classified into a number of molecular subtypes, namely (i) basal-like, also known as triple-negative BCa (TNBC) due to the absence of estrogen receptor (ER), progesterone receptor, and HER2; (ii) luminal A/B, characterized by the presence of ER; and finally, (iii) HER2 positive BCa, with amplified *ERBB2* gene expression [7, 8]. Clinically, these subtypes exhibit different responsiveness to chemotherapy and thus are administered different therapy regimens [9]. For example, luminal BCa is typically treated with chemotherapeutics that interfere with ER signaling, such as anti-estrogens and aromatase inhibitors [10]. Additionally, although controversial, different BCa subtypes exhibit distinct aberrations in their metabolic profiles [10, 11]. For instance, ER+ BCa has been shown to exhibit more “classical” Warburg metabolism, illustrated by an increase in glucose consumption and lactate production, whereas ER− BCa is known to rely more on glutamine metabolism and subsequent TCA replenishment/anaplerosis [10]. Moreover, metabolic adaptation has been reported as a potential resistance mechanism, which BCa cells adopt in response to hormone therapy [12]. Therefore, identification of drugs that target these altered metabolic networks may prove beneficial in the treatment of BCa as monotherapies, as well as enhance the efficacy/reduce resistance associated with currently established chemotherapeutics.

Recent work has shown that 1,25(OH)_2_D_3_ modulates glucose, glutamine, and fatty acid metabolism in several experimental models including breast and prostate cancer cells [13–17], which prompted us to thoroughly evaluate the ability of this molecule to regulate metabolic networks in BCa cell lines representing different molecular subtypes. The effect of 1,25(OH)_2_D_3_ on energy metabolism of luminal (MCF-7 and T-47D) and TNBC (MDA-MB-231, MDA-MB-468, and HCC-1143) cells was evaluated using real-time measurements of glycolytic/respiratory rates, GC/MS-based quantification of TCA cycle intermediates and diverse amino acids, mRNA expression analysis of metabolism-related genes, and finally overall energy charge. 1,25(OH)_2_D_3_ was found to induce both similar and different metabolic effects in these cell lines, such as induction in glucose-6-phosphate dehydrogenase (G6PD) expression and activity in all cell lines, and disparate regulation of glycolytic and respiratory capacities. In MCF-7 cells, seemingly pro-survival metabolic perturbations induced by treatment, such as accumulation of intracellular serine, were not found to antagonize the anti-tumor efficacy of chemotherapeutics including 5-fluorouracil (5-FU). Furthermore, 1,25(OH)_2_D_3_ was found to negatively regulate TXNIP expression in MCF-7 cells, possibly through reduction of estrogen receptor (ER) expression.

## Methods

### Cell culture

The human BCa cell lines MCF-7, T-47D, MDA-MB-231, MDA-MB-468, and HCC-1143 were cultured in Dulbecco’s Modified Eagle Medium (DMEM) (Gibco, Germany) containing 10% FCS (*v*/*v*) (Gibco, Germany) and 1% penicillin/streptomycin (*v*/*v*) (Gibco, Germany) and kept in a standard cell culture incubator (37 °C; 5% CO_2_). T-47D, MDA-MB-468, and HCC-1143 were kind gifts from Dr. Stefan Wiemann (DKFZ, Heidelberg). 1,25(OH)_2_D_3_ (Cayman Chemical-Biomol GmbH, Germany) treatments were performed for the various indicated time points in standard medium, at a final concentration of 100 nM. Additional drugs used in the study were added to either fresh or conditioned medium for different time points (as described in the “Results” section) and included 10 mM 2-deoxyglucose (Fluka-Sigma-Aldrich, Germany), 5 μM MG-132 (Sigma-Aldrich, Germany), 20 μM leupeptin (Sigma-Aldrich, Germany), 100 nM calcipotriol (Cayman Chemical-Biomol GmbH, Germany), and 20 μM BAPTA-AM (Cayman Chemical-Biomol GmbH, Germany), as well as increasing concentrations of dehydroepiandrosterone (DHEA) (Cayman Chemical-Biomol GmbH, Germany), Na Oxamate (Cayman Chemical-Biomol GmbH, Germany), AZD-3965 (Cayman Chemical-Biomol GmbH, Germany), 5-fluorouracil (5-FU) (Fluka-Sigma-Aldrich, Germany), and CBR-5884 (Cayman Chemical-Biomol GmbH, Germany).

### RNA isolation, cDNA synthesis, and RT-qPCR

BCa cells were treated with 1,25(OH)_2_D_3_ (100 nM) for 72 h, after which total RNA was isolated using QIAzol lysis reagent (Qiagen, Germany), following the manufacturer’s instructions. RNA concentration and purity were determined using NanoDrop 2000 UV-Vis Spectrophotometer (Thermo Scientific, Germany). cDNA was synthesized from 500 ng of total RNA/sample using ProtoScript® II first strand cDNA synthesis kit (New England BioLabs, Germany). The thermal cycle qTower (Analytik Jena AG) was used to assess mRNA expression of various genes in real time. Primers used in the study are listed in Additional file 1: Table S1. The LightCycler® 480 SYBR Green I Master (Roche, Germany) reaction mix was used. The ∆∆Ct method was used to calculate the relative expression of the investigated genes in response to treatment, with vinculin as the housekeeping gene.

### RNA interference

Anti-AMPKα1 and anti-G6PD siRNAs were synthesized by Riboxx (Riboxx GmbH, Radebeul, Germany) (Additional file 2: Table S2). MCF-7 cells were transfected with negative control, anti-AMPKα1, or anti-G6PD oligonucleotides as described in an earlier study [16]. Briefly, 50 pmol of the respective siRNA was diluted in 100 μL Opti-MEM Reduced Serum Medium (Gibco, Germany)/well in a 24-well plate and complexed with the 1.5 μL transfection reagent Lipofectamine® 3000 (Thermo Fischer, Germany)/well. Sixty thousand cells suspended in 500 μL of DMEM (10% FCS, without antibiotics) were added to each well and mixed gently. The plate was kept overnight in a standard incubator, and the treatment was performed on the following day as indicated.

### On-line measurement of extracellular acidification, oxygen consumption, and impedance using BIONAS 2500

Changes in cellular glycolytic, respiratory, and impedance rates were monitored in response to 1,25(OH)_2_D_3_ (100 nM) treatment using the Bionas 2500 biosensor chip system (Bionas, Rostock, Germany) as previously described [16, 18]. Briefly, 6 sensor chips (SC1000) were seeded with BCa cells at a density of 200,000 cells/chip in 450 μL of full medium and kept in a standard cell culture incubator overnight. The 6 chips were then transferred to the sensor chip system, where either 3 were continuously fed running medium (RM) containing either DMSO or 1,25(OH)_2_D_3_. The RM used was prepared from DMEM powder (Pan-Biotech GmbH, Germany) containing neither glucose, sodium pyruvate, L-glutamine, NaHCO3, nor phenol red. The medium was then supplemented with 1 g/L glucose, 2 mM glutamine, 1 mM HEPES, and 10 mg/L phenol red. Additionally, 0.1% (*v*/*v*) FCS and 1% (*v*/*v*) penicillin/streptomycin were added. Data were analyzed using the software provided by the manufacturer.

### Glucose uptake and ATP measurements

With regard to glucose uptake, culture medium was replaced with medium containing the 50 μM of the fluorescently labeled glucose analogue, 2-[N-(7-nitrobenz-2-oxa-1,3-diazol-4-yl) amino]-2-deoxy-D-glucose (2-NBDG) (Cayman Chemical-Biomol GmbH, Germany), at the end of the indicated treatment periods. Cells were harvested 2 h later, and FACS analysis was performed to determine the amount of intracellular 2-NBDG.

Intracellular ATP levels were assessed using the ATPlite™ 1 step (Perkin Elmer, Germany), following the manufacturer’s instructions. Cells were seeded at a density of 5000 cells/well in a black 96-well plate with a transparent bottom and placed inside a standard cell culture incubator overnight. The cells were then treated for 24 h with 100 μL of full medium containing either DMSO or 1,25(OH)_2_D_3_ (100 nM), after which 100 μL of the substrate solution was added to the wells, and luminescence was measured kinetically using the Tecan Ultra plate reader (Tecan, Germany). The signal obtained from each well was then normalized to the corresponding protein content.

### Analysis of glucose-6-phosphate dehydrogenase (G6PD) enzymatic activity

G6PD enzymatic activity was determined as previously described [19, 20]. The increase in absorbance resulting from the NADP+ to NADPH reduction was measured at 345 nm. NADPH is generated in the first and third reactions of the oxidative phase of the pentose phosphate pathway (PPP), namely the conversion of G6P to 6-phosphogluconolactone and 6-phosphogluconate to ribose-5-phosphate, catalyzed by G6PD and 6-phosphogluconate dehydrogenase, respectively. The combined activities of both enzymes were determined by obtaining crude extracts containing 500 ng total proteins from different cell lines treated for 72 h with either 1,25(OH)_2_D_3_ (100 nM) or DMSO and adding them to a solution containing 2 mM glucose-6-phosphate, 1 mM NADP+, and the reaction mixture (50 mM Tris/HCl buffer, pH 8.2, 100 mM KCl, 5 mM MgSO_4_), reaching a final volume of 100 μL. The activity of 6-phosphogluconate dehydrogenase was also measured by incubating 500 ng total proteins with 2 mM 6-phosphogluconate, 1 mM NADP+, and the aforementioned reaction mixture, reaching a final volume of 100 μL. The increase in absorbance obtained from both reactions was kinetically measured at 345 nm using the Tecan Ultra plate reader (Tecan, Germany), and the increase in absorbance associated with G6PD activity was determined as the different between the two reactions.

### Western blotting

After respective treatments were performed, cells were washed once with PBS and lysed with urea buffer containing a mixture of protease and phosphatase inhibitors (leupeptin, pepstatin, PMSF, aprotinin, sodium orthovanadate, and sodium pyrophosphate). Protein content in lysates was determined using Bradford assay (Sigma-Aldrich, Germany). Fifty micrograms of total proteins was resolved using SDS-PAGE and transferred onto a PVDF membrane (GE Healthcare, Germany). Membranes were blocked in blocking solution (5% milk in TBS/Tween) for 1 h at RT. Incubation with primary antibodies (diluted according to the manufacturer’s instructions in 5% BSA in TBS/Tween) was performed overnight at 4 °C. Anti-phosphorylated ACC (S79), total ACC, phosphorylated AMPK⍺ (T172), total AMPK⍺, ITCH, and ubiquitin (Ub) antibodies were obtained from Cell Signaling Technology; anti-TXNIP (VDUP1) antibody was purchased from MBL; anti-ß-actin, vinculin, and estrogen receptor (ER) ⍺ antibodies were purchased from Santa Cruz Biotechnology; and anti-G6PD antibody were purchased from Abgent. The membranes were incubated with anti-rabbit or anti-mouse IgG horseradish peroxidase-linked secondary antibodies (Cell Signaling Technology) for 1 h at RT, after which membranes were washed 3 times at RT using TBS/Tween. The horseradish peroxidase substrate Western Lightning™ Plus ECl (Perkin Elmer, Germany) was used, and target proteins were visualized using the Fujifilm LAS-3000 imaging system.

### Co-immunoprecipitation

MCF-7 cells were seeded in culture dishes until 80% confluency and then treated for 24 h with either DMSO or 1,25(OH)_2_D_3_. An additional control using MG-132 (6 h) was also used. Cells were then lysed as previously described, and samples were pre-cleared by incubating them with 0.25 mg of pre-washed protein A/G magnetic beads (Thermo Fischer, Germany) for 20 min at room temperature with rotation. The sample was then separated from the beads and incubated overnight with 2 μg of the antibody or an isotype specific control (Santa Cruz Biotechnology, Germany) at 4 °C with rotation. 0.25 mg of pre-washed protein A/G magnetic beads was then added to each sample, which was then incubated for 1 h at room temperature with rotation. Magnetic beads were then separated and then incubated with 100 μL of SDS-PAGE reducing buffer for 10 min at room temperature with rotation. Western blotting was then performed using the samples.

### Intracellular ROS measurements

Intracellular ROS levels in DMSO- and 1,25(OH)_2_D_3_-treated MCF-7 cells were assessed using dihydroethidium (DHE) (Biomol GmbH, Germany) and MitoSox Red (Thermo Fischer, Germany) staining. DHE is oxidized by superoxide to produce the fluorescent product oxyethidium, whereas the latter dye specifically detects mitochondrial superoxide. For DHE staining, MCF-7 cells were seeded in a μ-slide 8-well chamber (Ibidi, Germany) at a density of 30,000 cells/chamber and kept overnight in a standard cell culture incubator. Cells were then treated with DMSO or 1,25(OH)_2_D_3_ for 72 h, after which the medium was replaced with phenol red-free DMEM containing 2 μM Hoechst (Thermo Fischer, Germany), and cells were placed inside an incubator for 10 min. Medium was then replaced with phenol red-free DMEM containing 30 μM DHE and incubated for 15 mins, after which the medium was replaced with fresh phenol red-free DMEM, and images were taken using a fluorescence microscope (BZ9000, KEYENCE). Fluorescence intensity of DHE was quantified using ImageJ and normalized to DMSO-treated cells.

For MitoSox Red staining, MCF-7 cells were seeded in a 24-well plate at a density of 50,000 cells/well and treated for 72 h with either DMSO or 1,25(OH)_2_D_3_. Cells were then incubated with FCS-free DMEM containing 2 nM MitoTracker Green (Thermo Fischer, Germany) for 15 min. Medium was then replaced with fresh DMEM containing 5 μM MitoSox Red, and then, cells were incubated for 15 min. Medium containing the dye was then replaced with phenol red-free medium, and time-lapse images (every 15 min) were taken using IncuCyte ZOOM live cell analysis system (Essen BioScience, Germany). The Incucyte software 2016b was used to analyze the signal intensity based on a fluorescent area mask, with a top hat filter applied for dead cell exclusion due to enhanced auto-fluorescence. Four pictures per well were used to calculate the overall signal per well, and each condition was performed in 4 wells.

### Gas chromatography/mass spectrometry (GC/MS) analysis of metabolites/amino acids

#### Extraction

One hundred eighty microliters of 100% MeOH was added to frozen pellets, containing approximately 3.0 × 10^6^ cells, and subjected to vigorous shaking for 15 min at 70 °C. Ribitol was used as internal standard, and 5 μL of a 0.2 mg/ml stock was added to each sample. One hundred microliters of chloroform were then added, and samples were incubated for 5 min at 37 °C. Two hundred microliters of water were added to samples, which were then centrifuged at 11,000 g for 10 min to separate polar and organic phases. For derivatization, the polar phase (300 μL) was transferred to a new tube and dried using a speed-vac.

#### Derivatization (silylation and methoximation)

Twenty microliters of methoximation reagent containing 20 mg/ml methoxyamine hydrochloride (Sigma-Aldrich, Germany, 226,904) in pyridine (Sigma-Aldrich, Germany, 270,970) were used to dissolve the pellets. Samples were then incubated for 2 h at 37 °C with shaking. 32.2 μL of N-methyl-N-(trimethylsilyl)trifluoroacetamide (MSTFA; Sigma-Aldrich, Germany, M7891) and 2.8 μL of Alkane Standard Mixture (50 mg/ml C_10_–C_40_; Fluka-Sigma-Aldrich, Germany, 68,281) were also added to samples for silylation. Samples were incubated at 37 °C for 30 min, after which they were transferred to glass vials for GC/MS analysis.

#### GC/MS analysis

The GC/MS-QP2010 Plus (Shimadzu®) fitted with a Zebron ZB 5MS column (Phenomenex®; 30 m × 0.25 mm × 0.25 μm) was used. An injection temperature of 230 °C was used, and 1 μL of each sample was injected with split 10 mode. The temperature program was initiated with a hold at 40 °C for 1 min, followed by a ramp of 6 °C/min to 210 °C, another 20 °C/min ramp till 330 °C, and a bake-out at 330 °C for 5 min, with helium as a carrier gas with constant linear velocity. The MS was employed with ion source and interface temperatures of 250 °C. A scan range (m/z) of 40–700 with a 0.2 s event time was used. For data processing, the “GCMS solution” software (Shimadzu®) was utilized.

### SRB assay

The sulforhodamine B (SRB) assay was performed as previously described [21, 22]. Cells were seeded at a density of 5000 cells/well in 96-well culture plates and were treated with the different molecules using the indicated concentrations and treatment periods. Serine and glycine deprivation studies were performed using serine- and glycine-free DMEM supplemented with dialyzed FCS (10% *v*/*v*) and penicillin/streptomycin (1% *v*/*v*). Serine or glycine was added to the medium at a final concentration of 0.4 mM. Serine- and/or glycine-free medium was treated with either DMSO or 1,25(OH)_2_D_3_, and SRB assay was performed 72 h later. At the end of the treatment period, cells were fixed by adding 100 μL 10% trichloroacetic acid per well. Culture plates were incubated at 4 °C for 1 h, after which the plates were washed three times with water. After drying the plates, 100 μL 0.054% SRB solution (SRB powder (Santa Cruz Biotechnology) in 1% acetic acid) were added to each well and incubated for 1 h at RT. SRB solution was subsequently discarded, and wells were washed with 200 μL/well 1% acetic acid. After drying the plates, the SRB dye was solubilized using 10 mM Tris (pH 10.5), and the absorbance was measured at 535 nm using the Tecan Ultra plate reader (Tecan, Germany).

### Statistical analyses

Data were analyzed using Microsoft Excel and Graphpad Prism. Densitometric analysis was performed using the ImageJ software. Statistical significance of observed changes in a measured parameter between DMSO- and 1,25(OH)_2_D_3_-treated cells was determined using a Student’s *t* test. A *p* value less than or equal to 0.05, 0.01, and 0.001 is denoted on figures by *, **, and ***, respectively. Error bars represent SD. Available datasets of Affymetrix microarray profiling of breast tumors were used (www.kmplot.com) [23]. The median of G6PD expression (probe ID 202275_at) separated tumors into those with high- and low-G6PD expression. Logrank *p* values and the hazard ratio (HR) (95% confidence interval) are calculated.

## Results

### 1,25(OH)_2_D_3_ induces similar and disparate effects on glucose metabolism in different BCa cell lines

**Fig. 1:**
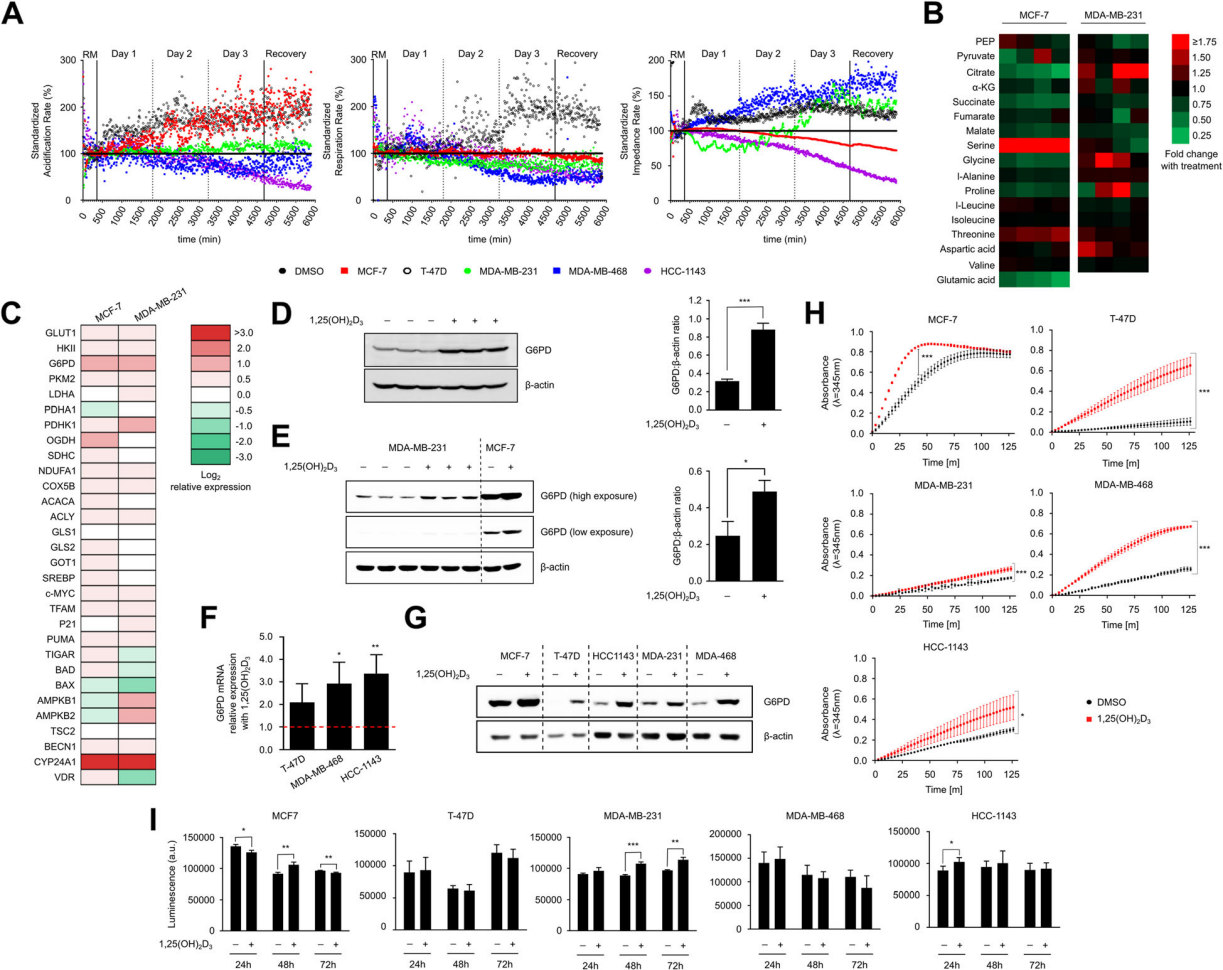
Analysis of 1,25(OH)_2_D_3_’s metabolism-regulating effects in BCa cells. **a** Extracellular acidification, respiration, and impedance rates were monitored in real-time in response to 1,25(OH)_2_D_3_ (100 nM) over the course of 3 days, followed by a 20-h recovery period, in which cells were exposed to running medium (RM) not containing the molecule. 1,25(OH)_2_D_3_ clearly induces glycolytic rate in luminal (MCF-7 and T-47D) BCa cells and reduces respiration rate to varying degrees in TNBC (MDA-MB-231, MDA-MB-468, and HCC-1143) cells. Values of each cell line are normalized to measurements obtained from the respective DMSO-treated cells. Data presented are representative of 2 biological replicates. **b** GC/MS analysis of TCA cycle intermediates and amino acids in select BCa cell lines treated for 72 h with 1,25(OH)_2_D_3_ (*n* = 4). A strong accumulation in serine is observed with treatment in MCF-7 cells, whereas levels of citrate were differentially regulated by 1,25(OH)_2_D_3_ in both cell lines. PEP phosphoenolpyruvate, α-KG alpha-ketoglutarate. **c** mRNA expression analysis of metabolism-related genes in MCF-7 and MDA-MB-231 cells in response to a 72 h treatment with 1,25(OH)_2_D_3_. G6PD was found to be induced by 1,25(OH)_2_D_3_ in both cell lines. Relative expression was calculated using the ∆∆Ct method, with vinculin as the housekeeping gene. Data presented are the average of 2 biological replicates. G6PD protein expression is significantly induced in MCF-7 (**d**) and MDA-MB-231 (**e**) cells by 1,25(OH)_2_D_3_ (72 h), as demonstrated by western blot and densitometric analysis. Statistical comparison between DMSO- and 1,25(OH)_2_D_3_-treated cells was made using a two-tailed Student’s *t* test. *P* values less than or equal to 0.05, 0.01 and 0.001 are depicted by *, **, and ***, respectively. Error bars ± SD; *n* = 3. G6PD mRNA (**f**), protein (**g**), and activity (**h**) levels are induced by 1,25(OH)_2_D_3_ (72 h) in all BCa cell lines. **i** Intracellular ATP levels were assessed in BCa cells in response to 1, 2, and 3 days of treatment with 1,25(OH)_2_D_3_. Treatment significantly induced ATP levels in MDA-MB-231 cells in 48 and 72 h, however reduced ATP levels in MCF-7 at the latest time point

As previously mentioned, several recent studies have demonstrated that 1,25(OH)_2_D_3_ induces metabolic changes in different cancer models including BCa. To confirm this in the BCa cell lines included in this study, we employed a biosensor chip system that measures in real time, changes in extracellular acidification, oxygen consumption, and impedance. We observed clear differences in the metabolic response of all cell lines to 1,25(OH)_2_D_3_ treatment. In luminal breast cancer cells (MCF-7 and T-47D cells), 1,25(OH)_2_D_3_ markedly induced the acidification rate gradually over the investigated time course, but did not significantly impact the respiratory rate (Fig. 1a). On the other hand, 1,25(OH)_2_D_3_ was found to reduce respiration—to varying degrees—in TNBC cells (Fig. 1a).

To further investigate the influence of treatment on glucose metabolism, we studied the regulation of TCA cycle intermediates by 1,25(OH)_2_D_3_ using GC/MS in representative cell lines of each molecular subtype, MCF-7 (luminal) and MDA-MB-231 (TNBC) cells. Interestingly, the levels of the investigated intermediates were differentially impacted by 1,25(OH)_2_D_3_, where treatment was found to reduce citrate levels and the levels of downstream metabolites in MCF-7 cells (Fig. 1b). In MDA-MB-231 cells on the contrary, citrate levels were found to be increased by 1,25(OH)_2_D_3_ (Fig. 1b). Additionally, we investigated the levels of select essential and non-essential amino acids in response to 1,25(OH)_2_D_3_ in both cell lines. While levels of all investigated amino acids were altered in both cell lines by 1,25(OH)_2_D_3_ treatment to varying degrees, a profound increase in serine levels was observed in MCF-7 cells (Fig. 1b). Additionally, glycine levels were found to be reduced and increased in response to 1,25(OH)_2_D_3_ in MCF-7 and MDA-MB-231 cells, respectively (Fig. 1b).

In view of the effects 1,25(OH)_2_D_3_ exerted on the investigated metabolic parameters in BCa cells, we postulated that such effects may at least in part be explained by 1,25(OH)_2_D_3_-mediated changes in the expression of metabolism-related genes. We thus performed a comprehensive mRNA screen of genes encoding players involved in diverse metabolic processes, such as glucose, glutamine, and fatty acid metabolism, in MCF-7 and MDA-MB-231 cells. Furthermore, given the different p53 backgrounds exhibited by the two investigated cell lines, and since p53 is known to be a powerful regulator of cell metabolism and associated processes (e.g., autophagy) [24, 25], we included in our screen a number of p53 target genes, as well as genes encoding autophagy regulators. While several investigated genes were only mildly regulated by 1,25(OH)_2_D_3_ treatment in both MCF-7 and MDA-MB-231 cells, a clear induction in the mRNA levels of the *G6PD* gene, encoding glucose-6-phosphate dehydrogenase, was observed in both cell lines (Fig. 1c). Similarly, a significant induction in G6PD protein expression was observed with 1,25(OH)_2_D_3_ treatment in both cell lines (Fig. 1d, e). G6PD catalyzes the first rate-limiting step in the PPP, which provides cells with reducing equivalents for both reductive biosynthesis and protection against oxidative stress [26]. On the other hand, G6PD expression has been shown to be elevated in cancers and its activity induced by oncogenic signaling [26, 27]. With regard to vitamin D, the promoter of the *G6PD* gene has been shown to harbor vitamin D response elements [28], and 1,25(OH)_2_D_3_ has been repeatedly shown to induce either the expression and/or activity of this enzyme in different experimental models, including BCa [29, 30]. We then aimed to investigate if the upregulation of G6PD expression with 1,25(OH)_2_D_3_ treatment is observed in the other BCa cell lines. Indeed, G6PD mRNA and protein levels were markedly induced with 1,25(OH)_2_D_3_ in all the investigated BCa cell lines (Fig. 1f, g). Importantly, G6PD enzymatic activity in all cell lines was found to be significantly increased in response to treatment (Fig. 1h).

We also sought to investigate changes in cellular ATP levels in response to 1,25(OH)_2_D_3_ treatment in all BCa cell lines. In MCF-7 cells, 1,25(OH)_2_D_3_ was found to induce fluctuations in cellular ATP levels over the investigated time course, with an overall significant decrease with treatment after 72 h (Fig. 1i). In MDA-MB-231 cells, no clear differences in ATP levels were observed with treatment after 24 h; however, a profound induction was observed after 48 and 72 h (Fig. 1i).

### Seemingly pro-survival, 1,25(OH)_2_D_3_-induced metabolic changes do not hamper the efficacy of different anti-cancer strategies

**Fig. 2:**
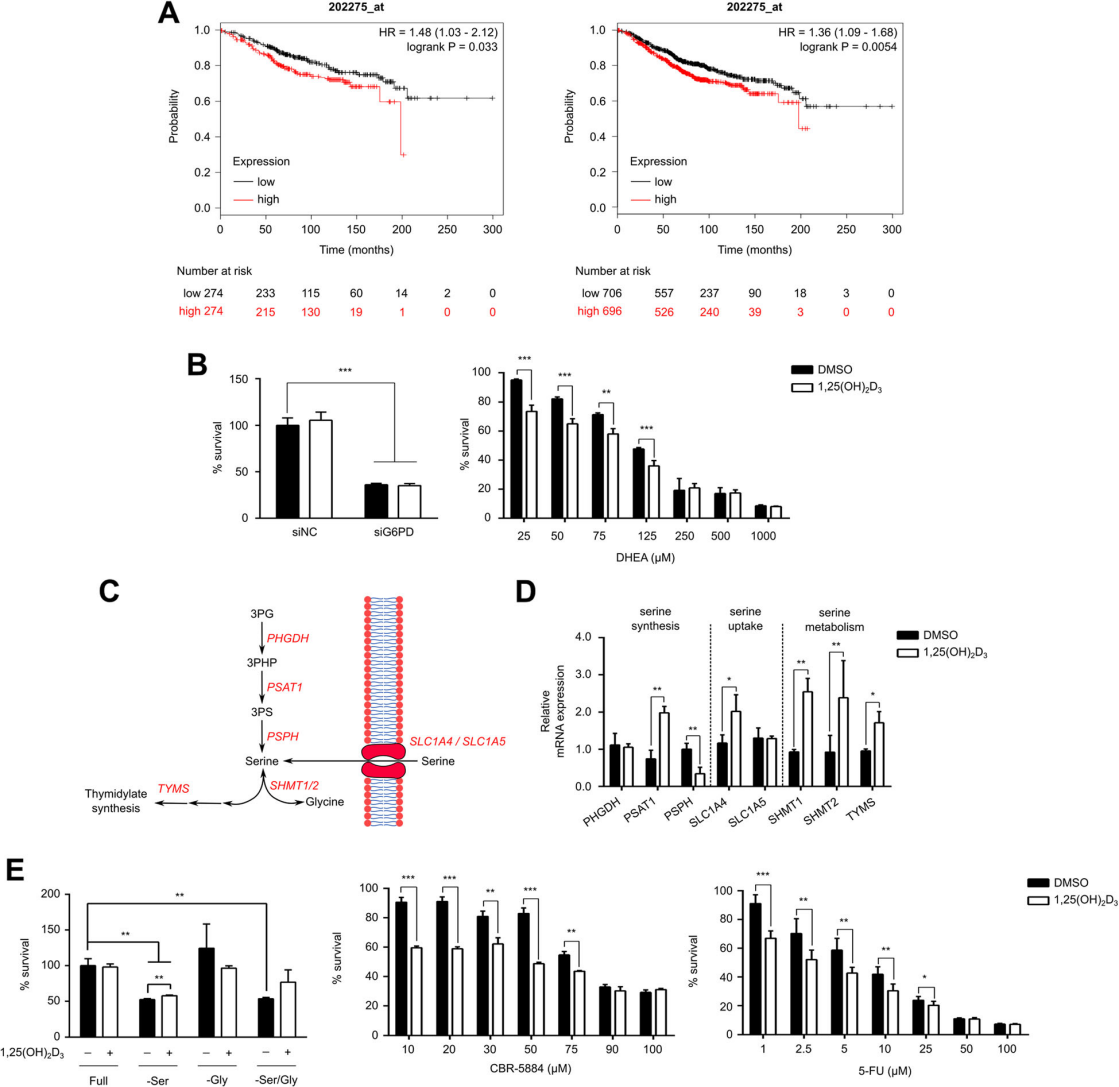
Seemingly pro-survival metabolic effects of 1,25(OH)_2_D_3_ do not hamper the efficacy of metabolism-targeting therapeutic regimens. **a** Kaplan-Meier plots demonstrating the significant inverse correlation between G6PD mRNA expression and overall survival in ER+ (left) and total (right) BCa patients. **b** Knocking-down G6PD in MCF-7 cells significantly reduces cell survival, independent of 1,25(OH)_2_D_3_ (100 nM; 72 h). Statistical comparison between DMSO- and 1,25(OH)_2_D_3_-treated cells was made using a two-tailed Student’s *t* test. *P* values less than or equal to 0.05, 0.01 and 0.001 are depicted by *, **, and ***, respectively. Error bars ± SD; *n* = 3. Cellular survival was assessed using SRB assay, and % survival is calculated by normalizing the absorbance value obtained with the different experimental conditions to DMSO-treated cells transfected with negative control (NC) siRNA. 1,25(OH)_2_D_3_ significantly enhances the anti-tumor effects of DHEA assessed by SRB assay. MCF-7 cells were treated for 72 h with increasing concentrations of DHEA in the presence of either DMSO or 1,25(OH)_2_D_3_, and % survival is calculated by normalizing the absorbance value obtained with the different experimental conditions to DMSO-treated cells. **c** Schematic overview of cellular serine uptake, synthesis, and metabolism. 3PS 3-phosphoserine. **d** 72 h treatment of MCF-7 cells with 1,25(OH)_2_D_3_ differentially influences the mRNA expression of enzymes involved in serine synthesis but upregulates the expression of those involved in serine metabolism. Relative expression was calculated using the ∆∆Ct method, with vinculin as the housekeeping gene. **e** 1,25(OH)_2_D_3_ significantly influences cellular survival in response to amino acid deprivation. MCF-7 cells were cultivated in medium lacking serine, glycine, or both, in the presence of either DMSO or 1,25(OH)_2_D_3_ for 72 h, after which SRB assay was performed. 1,25(OH)_2_D_3_ was found to mildly however significantly dampen the reduction in survival of MCF-7 cells in response to serine deprivation. 1,25(OH)_2_D_3_ was also found to enhance the efficacy of CBR-5884 and 5-FU, inhibitors of PHGDH and TYMS, respectively, assessed by SRB assay

Results of the metabolic characterization described in the previous section indicate that certain effects induced by 1,25(OH)_2_D_3_ might enhance cancer cell proliferation and survival. For example, in MCF-7 cells, treatment was found to increase intracellular serine levels and G6PD expression and activity. G6PD, as previously mentioned, is a putative oncogene, whose expression significantly correlates with reduced overall survival in ER+ and total BCa patients (Fig. 2a). Similarly, serine is a recognized onco-metabolite that is known to contribute to both the folate and methionine cycles, thus enhancing nucleotide biosynthesis, NADPH production, and S-adenosylmethionine generation, possibly inducing epigenetic changes [31].

G6PD inhibition has been previously shown to limit cancer cell survival [32]. To investigate if 1,25(OH)_2_D_3_’s induction of G6PD expression and activity would hamper such anti-tumor strategies, G6PD was genetically and pharmacologically inhibited in MCF-7 cells using siRNA and DHEA, respectively, in the presence and absence of 1,25(OH)_2_D_3_, and cellular survival was assessed by SRB assay. While 1,25(OH)_2_D_3_ treatment did not dampen the significant reduction in cell survival in response to G6PD knockdown, the inhibitory (anti-cancer) effect of DHEA was found to be enhanced in the presence of 1,25(OH)_2_D_3_ (Fig. 2b).

We then aimed at investigating the underlying mechanism of serine accumulation in response to 1,25(OH)_2_D_3_ in MCF-7 cells. Serine is a non-essential amino acid that cells acquire either by de novo synthesis, using the glycolytic intermediate 3-phosphoglycerate (3PG) as a starting molecule, or by uptake from the medium (Fig. 2c). We therefore investigated changes in the mRNA expression of players involved in serine synthesis, metabolism, and uptake, in response to 1,25(OH)_2_D_3_ treatment of MCF-7 cells. While treatment was found to differentially regulate the mRNA expression of enzymes in the serine synthesis pathway (SSP), for example non-significant alteration of PHGDH (phosphoglycerate dehydrogenase), induction of PSAT1 (phosphoserine aminotransferase 1), and reduction of PSPH (phosphoserine phosphatase), enzymes involved in serine metabolism, namely serine hydroxymethyltransferase 1 (SHMT1) and 2 (SHMT2), as well as thymidylate synthase (TYMS), were found to be significantly induced with 1,25(OH)_2_D_3_ (Fig. 2d). SHMT1 and SHMT2 are responsible for the interconversion of serine and glycine, and, concurrently, tetrahydrofolate (THF) and 5,10-methylene-THF [31]. The latter could be used in the synthesis of thymidylate, in a reaction catalyzed by TYMS, which is the intracellular target of the classical chemotherapeutic 5-fluorouracil (5-FU) [33].

To investigate the impact of serine accumulation in 1,25(OH)_2_D_3_-treated MCF-7 cells on the efficacy of 5-FU, cells were treated with increasing concentrations of 5-FU, alone and in combination with 1,25(OH)_2_D_3_, and cellular survival was evaluated. The addition of 1,25(OH)_2_D_3_ was found to significantly improve 5-FU’s anti-cancer effects (Fig. 2e). We then aimed to elucidate whether serine accumulation was the result of increased de novo synthesis or uptake. We therefore treated MCF-7 cells with the small molecule PHGDH inhibitor CBR-5884 in the presence and absence of 1,25(OH)_2_D_3_. PHGDH catalyzes the first reaction of the SSP, namely the conversion of 3PG to 3-phosphopyruvate (3PHP) [31]. CBR-5884 has been shown to be toxic to cancer cells that depend on de novo serine synthesis, but not to cancer cells reliant on extracellular sources for serine [34]. Mullarky et al. [34] demonstrated that MCF-7 cells are dependent on serine in the medium and were therefore insensitive to CBR-5884. However, upon cultivation in serine-depleted medium, MCF-7 cells were rendered susceptible to the drug’s toxic effects [34]. We thus postulated that an increased SSP activity in response to 1,25(OH)_2_D_3_ would render MCF-7 sensitive to CBR-5884’s anti-tumor effects on the one hand, as well as dampen the growth inhibiting effects of serine deprivation on the other. Indeed, treating MCF-7 cells with a combination of 1,25(OH)_2_D_3_ and increasing concentrations of CBR-5884 led to substantial inhibition of proliferation compared to cells treated with CBR-5884 alone (Fig. 2e). Additionally, 1,25(OH)_2_D_3_ was found to mildly however significantly reduce the sensitivity of MCF-7 cells to serine deprivation. Altogether, these results indicate that 1,25(OH)_2_D_3_ may enhance the ability of cells to synthesize serine. It remains unclear, however, if the proposed increase in serine synthesis utilizes the glycolytic metabolite 3PG or glycine as a starting intermediate. In support of the latter possibility is the reduction in glycine levels and the induction in SHMT1 and 2 mRNA expression in 1,25(OH)_2_D_3_-treated MCF-7 cells.

### 1,25(OH)_2_D_3_ differentially regulates the AMPK-TXNIP signaling axis in BCa cells

In view of the clear metabolic rewiring induced by 1,25(OH)_2_D_3_ in BCa cells, we hypothesized that key signaling molecules involved in regulating energy levels and glucose homeostasis might be affected by treatment. We thus investigated changes in TXNIP expression and AMPK signaling activation in BCa cells in response to 1,25(OH)_2_D_3_ treatment. TXNIP, originally identified by Chen and DeLuca in HL-60 cells as the vitamin D upregulated protein 1 (VDUP1) [35], regulates both redox balance and glucose uptake [36]. With regard to redox regulation, TXNIP binds to reduced thioredoxin and prevents its anti-oxidant actions [36]. More recent studies however have shown that TXNIP is a pivotal member of glucose-sensing mechanisms, involving glycolytic intermediates, glucose transporters, and the heterodimers MondoA-MLX [37, 38]. Briefly, an increase in glucose uptake and subsequently glycolytic intermediates triggers the nuclear translocation of the aforementioned heterodimer, which binds to carbohydrate response elements on the *TXNIP* gene, thereby inducing its expression [37]. TXNIP in turn reduces glucose uptake by reducing the expression and inducing the internalization of glucose transporter 1 [38]. AMPK on the other hand is a heterotrimeric complex that acts as an intracellular energy gauge through sensing changes in the AMP:ATP ratio [39]. In response to energetic stress, AMPK inhibits energy-consuming processes such as fatty acid synthesis and activates energy-producing processes, including glucose uptake and mitochondrial respiration [39]. Recently, AMPK has been shown to induce glucose uptake through triggering the degradation of TXNIP [38].

**Fig. 3:**
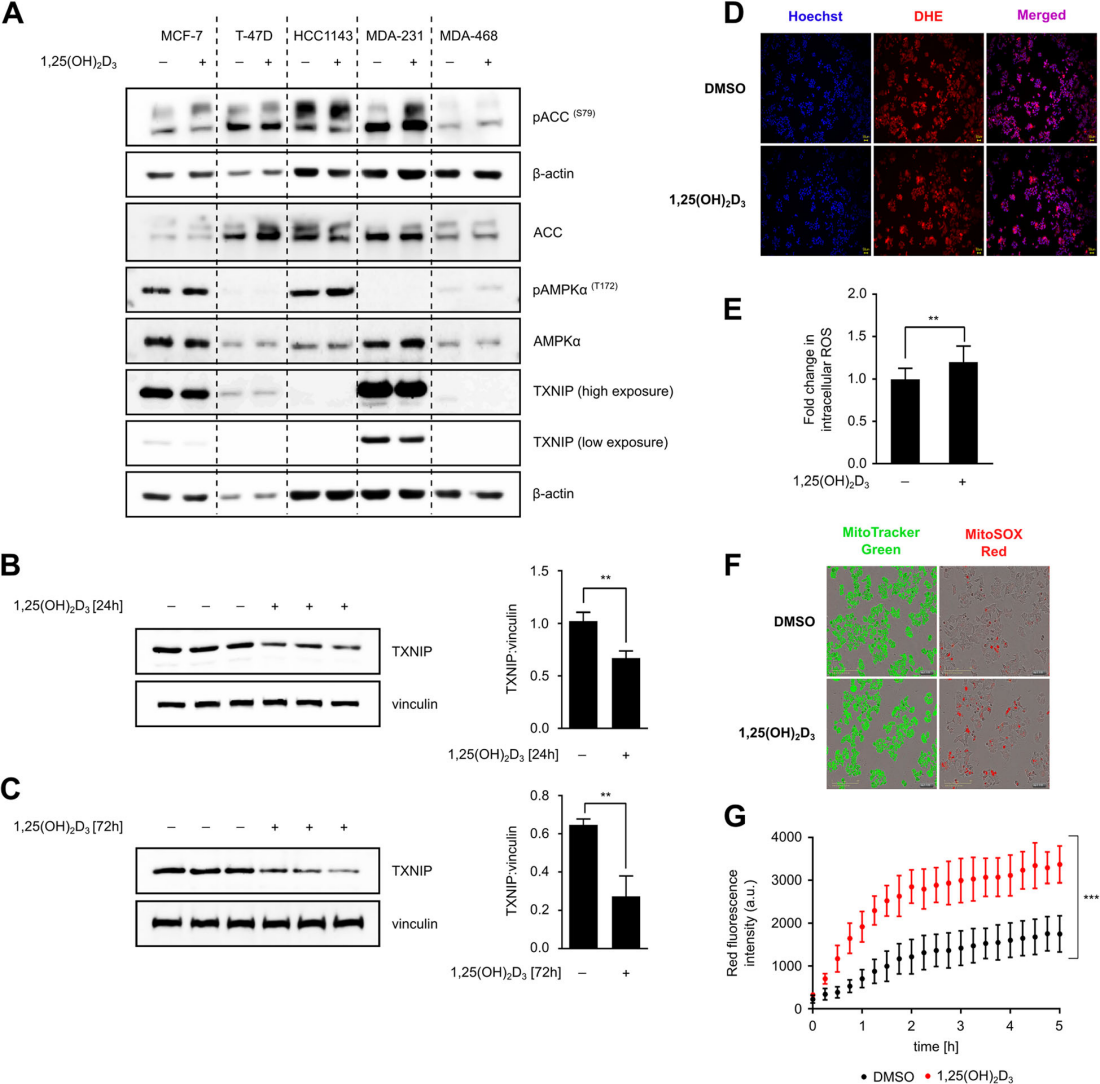
Regulation of AMPK signaling and TXNIP expression in BCa cells by 1,25(OH)_2_D_3_. **a** The AMPK-TXNIP signaling axis was found to be differentially regulated by a 72-h treatment with 1,25(OH)_2_D_3_ (100 nM). AMPK signaling was found to be induced and TXNIP expression reduced in MCF-7 cells in response to treatment. Western blot and densitometric analysis of TXNIP expression in MCF-7 cells treated for 24 (**b**) and 72 h (**c**) with 1,25(OH)_2_D_3_. Statistical significance between DMSO- and 1,25(OH)_2_D_3_-treated cells is calculated using a two-tailed Student’s *t* test, where *p* values less than or equal to 0.05, 0.01, and 0.001 are depicted in the figures by *, **, and ***, respectively. Error bars ± SD; *n* = 3. **d**–**g** 72 h treatment with 1,25(OH)_2_D_3_ significantly induces intracellular ROS levels. Microscopic analysis of cellular superoxide using DHE staining (**d**) and associated quantification (**e**). Hoechst dye was used to stain nuclei. Mitochondrial superoxide was stained using MitoSOX Red, and mitochondria were stained using MitoTracker Green (**f**). Microscopic images were taken every 15 min for 5 h, and fluorescence intensity of MitoSOX Red was quantified (**g**)

BCa cells were therefore treated with 1,25(OH)_2_D_3_ for 72 h, after which TXNIP levels and AMPK signaling activity were investigated using immunoblotting. Treatment was found to activate AMPK signaling, as observed by the increased phosphorylation of acetyl coA carboxylase (ACC; serine 79)—AMPK’s substrate—in MCF-7 and MDA-MB-231 cells (Fig. 3a), which correlates well with the alterations in ATP levels observed in these two cell lines with 1,25(OH)_2_D_3_ treatment (Fig. 1i). Furthermore, in cell lines expressing detectable levels of TXNIP, namely MCF-7, T-47D, and MDA-MB-231 cells, canonical regulation of TXNIP expression by 1,25(OH)_2_D_3_ was not observed (Fig. 3a). In fact, treatment was found to reduce TXNIP levels in MCF-7 cells (Fig. 3a), an effect that was statistically significant after both 24 and 72 h of treatment (Fig. 3b,  c). This is in line with our previous studies demonstrating reduction of TXNIP levels by 1,25(OH)_2_D_3_ in prostate cancer cells [16], as well as the cell line-specific regulation of the protein by treatment [40].

Since TXNIP engages in both redox balance and glucose homeostasis [37, 41], we hypothesized that the reduction in TXNIP level in MCF-7 cells by treatment may also influence such critical cellular processes. We therefore evaluated intracellular ROS levels in DMSO- and 1,25(OH)_2_D_3_-treated MCF-7 cells using DHE and MitoSOX Red staining. Although 1,25(OH)_2_D_3_ was found to significantly induce G6PD expression and activity (Fig. 1d, h) as well as intracellular serine levels in MCF-7 cells (Fig. 1c), which together with the reduction in TXNIP expression serve as a strong basis for anti-oxidant activity, we observed significant ROS accumulation in response to treatment (Fig. 3d–g; Additional files 3 and 4: Movies S1 and S2). This is in line with the results of Koren et al. [42], who illustrated that 1,25(OH)_2_D_3_ may act as a pro-oxidant in MCF-7 cells by increasing the ratio of oxidized to reduced glutathione. Moreover, we observed a significant reduction in glucose uptake after 24, but not 72 h of treatment (Additional file 5: Figure S1), indicating that the reduction in intracellular glycolytic intermediates in response to 1,25(OH)_2_D_3_ may have led to the decrease in TXNIP expression.

### Non-canonical regulation of TXNIP by 1,25(OH)_2_D_3_ is independent of metabolic changes

**Fig. 4:**
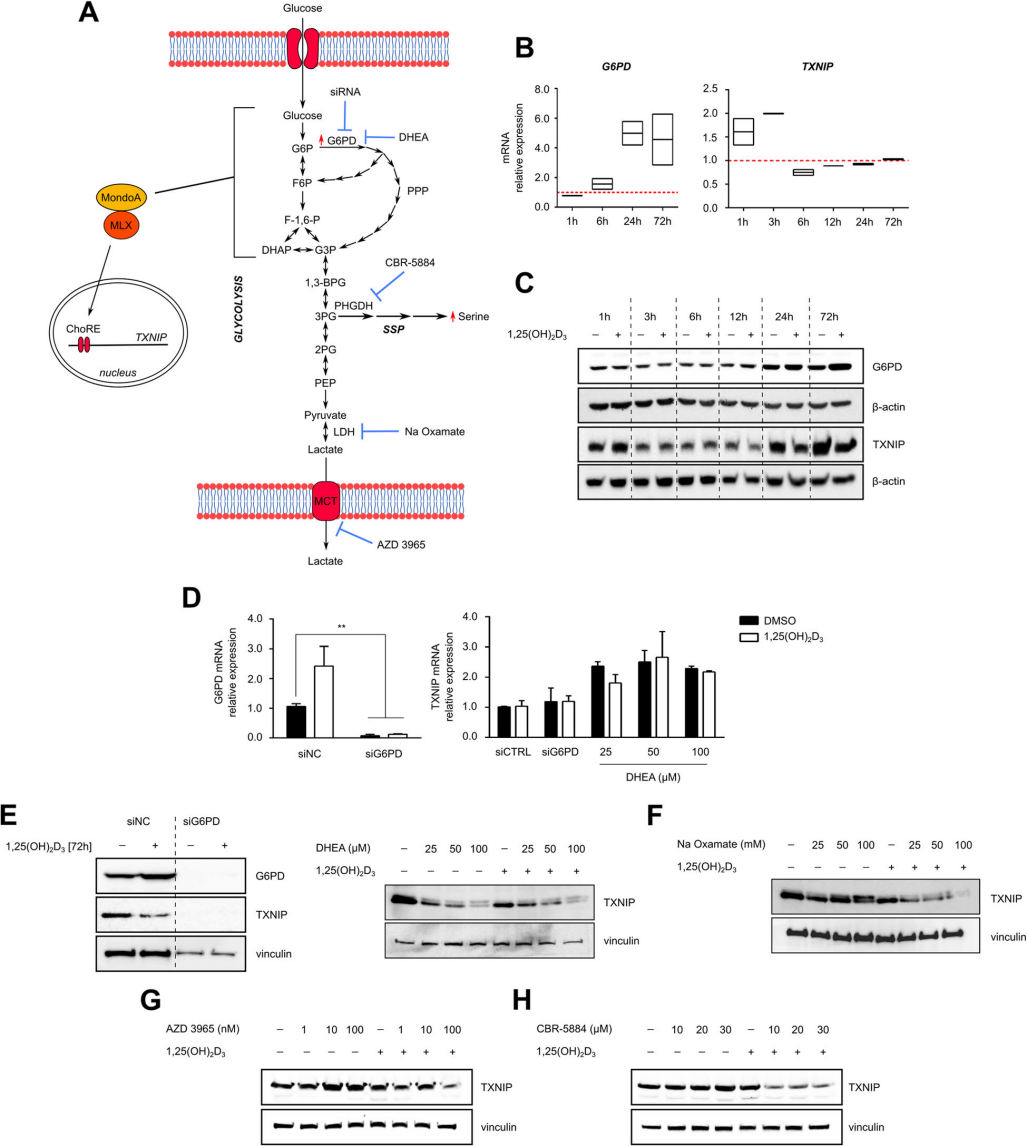
TXNIP regulation by 1,25(OH)_2_D_3_ is independent of metabolic reprogramming. **a** Schematic overview of treatment-induced metabolic changes that may reduce levels of glycolytic intermediates capable of inducing the nuclear translocation of MondoA/MLX and hence TXNIP expression. Genetic and/or pharmacological strategies for inhibiting key metabolic players are depicted. **b** Time-dependent increase in G6PD mRNA expression in MCF-7 cells with 1,25(OH)_2_D_3_ (100 nM) is associated with an attenuation of the initial increase in TXNIP mRNA levels with treatment. Upper and lower ends of floating bars represent maximum and minimum values, respectively, whereas lines in the middle represent the means. The ∆∆Ct method was used to calculate the relative expression with vinculin as the housekeeping gene. The data are obtained from two biological replicates. **c** Similar to the trend observed on the mRNA level, the induction in G6PD protein level in response to 1,25(OH)_2_D_3_ first occurs after 12 h, which coincides with the first reduction in TXNIP protein expression. **d** Co-treatment with DHEA, but not knocking-down G6PD levels, elevates TXNIP mRNA levels in 1,25(OH)_2_D_3_-treated MCF-7 cells. Cells transfected with either anti-G6PD siRNA or a NC were treated with 1,25(OH)_2_D_3_ for 72 h, whereas increasing concentrations of DHEA were added to the conditioned medium of MCF-7 cells treated for 48 h with either DMSO or 1,25(OH)_2_D_3_, for an additional 24 h. **e** G6PD inhibition using siRNA or DHEA reduces TXNIP protein expression independent of 1,25(OH)_2_D_3_. **f**–**h** Western blot analysis of TXNIP expression in MCF-7 cells treated with a combination of either DMSO or 1,25(OH)_2_D_3_ and various metabolic inhibitors. Cells were initially treated with DMSO or 1,25(OH)_2_D_3_ for 48 h, after which increasing concentrations of the different inhibitors were added to the conditioned medium for an additional 24 h. All inhibitors were found to reduce TXNIP protein levels in 1,25(OH)_2_D_3_-treated cells. In the absence of 1,25(OH)_2_D_3_, AZD 3965 and CBR-5884 were found to mildly induce TXNIP levels

As previously mentioned, the *TXNIP* gene is a direct target of the glucose-sensing transcriptional heterodimer MondoA-MLX and is induced in response to an increase in upstream glycolytic intermediates, namely glucose-6-phosphate. We postulated that distinct metabolic changes induced by 1,25(OH)_2_D_3_ in MCF-7 cells, including increased G6PD expression and activity, induction of the SSP, and enhanced glycolytic rate, may contribute to the reduction in TXNIP expression by depleting the levels of glycolytic intermediates capable of inducing TXNIP levels. We thus postulated that genetic and/or pharmacological inhibition of such metabolic pathways in 1,25(OH)_2_D_3_-treated cells would rescue TXNIP expression (Fig. 4a).

Since G6P is a major glycolytic metabolite implicated in inducing TXNIP expression [43], we first investigated the impact of G6PD induction by 1,25(OH)_2_D_3_ on TXNIP regulation. Time-course studies of TXNIP and G6PD mRNA and protein regulation by 1,25(OH)_2_D_3_ illustrated that the initial induction in TXNIP mRNA levels by treatment (1–3 h) was found to be largely attenuated at 6 h, coinciding with the induction in G6PD mRNA levels by 1,25(OH)_2_D_3_ (Fig. 4b). Similarly, on the protein level, the reduction and induction of TXNIP and G6PD levels, respectively, in response to treatment, first occurs after 12 h (Fig. 4c), indicating that shunting of G6P into the PPP by G6PD may be responsible for TXNIP reduction. We then knocked-down G6PD levels in MCF-7 cells using siRNA and investigated TXNIP mRNA and protein regulation by 1,25(OH)_2_D_3_. Additionally, cells were treated with either DMSO or 1,25(OH)_2_D_3_ for 48 h, and DHEA—the non-competitive inhibitor of G6PD [44]—was added to the conditioned medium for an additional 24 h, after which TXNIP levels were also investigated. Independent of 1,25(OH)_2_D_3_, knocking-down G6PD levels were not found to influence TXNIP mRNA levels, whereas different concentrations of DHEA were found to induce TXNIP mRNA levels (Fig. 4d). Furthermore, TXNIP protein expression was found to be largely depleted, and reduced in a dose-dependent manner, in cells with knocked-down G6PD levels, and in DHEA-treated cells, respectively, independent of 1,25(OH)_2_D_3_ (Fig. 4e). We therefore concluded that the induction in G6PD expression and activity by treatment does not contribute to the reduction in TXNIP expression.

To assess the contribution of other 1,25(OH)_2_D_3_-induced metabolic changes to TXNIP regulation, MCF-7 cells were treated with increasing concentrations of Na oxamate and AZD 3965, inhibitors of lactate dehydrogenase and monocarboxylate transporter (MCT) 1, respectively, as well as CBR-5884, alone and in combination with 1,25(OH)_2_D_3_. While AZD 3965 and CBR-5884 led to slight increases in TXNIP protein levels, all inhibitors were found to reduce TXNIP expression in the presence of 1,25(OH)_2_D_3_ (Fig. 4f–h), indicating that inhibition of one metabolic pathway in 1,25(OH)_2_D_3_-treated cells does not lead to an accumulation of glycolytic intermediates since such metabolites might be shunted into other activated pathways.

### 1,25(OH)_2_D_3_ reduces TXNIP expression through inducing its proteasomal degradation

Since 1,25(OH)_2_D_3_ treatment had no profound impact on TXNIP mRNA levels at time points in which protein levels were found to be clearly reduced by treatment (12, 24, and 72 h), we speculated that 1,25(OH)_2_D_3_ reduces TXNIP levels through inducing its proteasomal degradation. Numerous studies have demonstrated that 1,25(OH)_2_D_3_ influences protein stability through a number of distinct mechanisms, namely regulating the expression of players in the ubiquitin proteasomal pathway, proteases, and protease inhibitors [45]. Moreover, TXNIP degradation through the ubiquitin proteasomal pathway has been demonstrated by studies implicating the E3 ubiquitin ligase ITCH as well as AMPK signaling in TXNIP stability [38, 46].

**Fig. 5:**
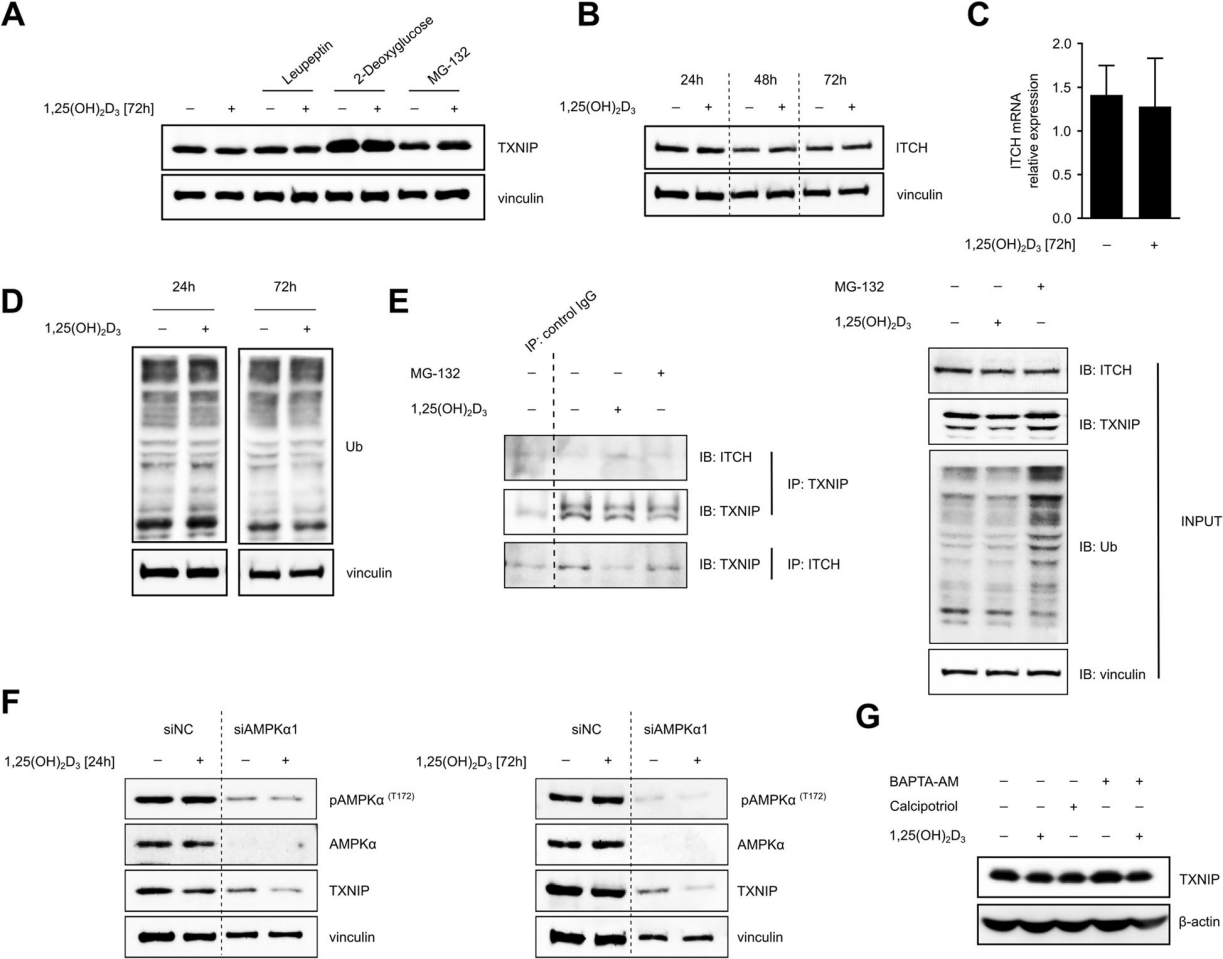
1,25(OH)_2_D_3_ induces proteasomal degradation of TXNIP in MCF-7 cells. **a** The reduction in TXNIP protein levels by 1,25(OH)_2_D_3_ (100 nM) in MCF-7 cells is rescued by MG-132 (5 μM) or 2-deoxyglucose (10 mM), but not leupeptin (20 μM). The various molecules were added to the conditioned medium of DMSO- and 1,25(OH)_2_D_3_-treated (66 h) MCF-7 cells, for an additional 6 h. **b**, **c** ITCH mRNA and protein expression is not markedly influenced by 1,25(OH)_2_D_3_. Relative expression was calculated using the ∆∆Ct method with vinculin as the housekeeping gene. Error bars ± SD; *n* > 3. **d** Overall protein ubiquitination in MCF-7 cells was not changed by 1,25(OH)_2_D_3_ treatment. **e** Co-immunoprecipitation studies illustrate that the TXNIP-ITCH interaction is not altered by 1,25(OH)_2_D_3_ treatment of MCF-7 cells. **f** Negative regulation of TXNIP protein expression by 1,25(OH)_2_D_3_ is observed in MCF-7 cells with knocked-down AMPKα1 levels. **g** The non-calcemic 1,25(OH)_2_D_3_ analogue, calcipotriol (100 nM; 72 h) induces similar effects on TXNIP expression as 1,25(OH)_2_D_3_. The cell permeable Ca^2+^ chelator BAPTA-AM (20 μM) does not hamper 1,25(OH)_2_D_3_’s effects on TXNIP expression. BAPTA-AM was added to the conditioned medium of DMSO- and 1,25(OH)_2_D_3_-treated MCF-7 cells, 2 h prior to the end of the treatment period (72 h)

To investigate the possible degradation of TXNIP by 1,25(OH)_2_D_3_, the proteasomal and lysosomal inhibitors MG-132 and leupeptin, respectively, were added to the conditioned medium of DMSO- and 1,25(OH)_2_D_3_-treated MCF-7 cells 6 h prior to the end of the treatment period (72 h). A similar treatment plan was employed for 2-deoxyglucose, which was used as a positive control of TXNIP regulation, since this molecule is phosphorylated by hexokinase to yield 2-deoxyglucose-6-phosphate, which accumulates intracellularly, thus inducing the nuclear translocation of MondoA/Mlx leading to an increase in TXNIP expression [37]. MG-132 and 2-deoxyglucose, but not leupeptin, were found to rescue TXNIP levels in 1,25(OH)_2_D_3_-treated cells (Fig. 5a).

In view of this, we proposed that 1,25(OH)_2_D_3_ may influence TXNIP proteasomal degradation by either inducing ITCH expression or by enhancing its interaction with TXNIP. Assessment of changes in ITCH mRNA and protein levels in MCF-7 cells in response to 1,25(OH)_2_D_3_ did not reveal an induction in ITCH expression by treatment (Fig. 5b, c). Similarly, overall protein ubiquitination in MCF-7 cells was unchanged by 1,25(OH)_2_D_3_ treatment (Fig. 5d). Furthermore, 1,25(OH)_2_D_3_ was not found to markedly alter the levels of ITCH co-precipitating with TXNIP (Fig. 5e). We then hypothesized that AMPK, which was found to be activated in response to 1,25(OH)_2_D_3_ in MCF-7 cells (Fig. 3a), may be implicated in TXNIP degradation by 1,25(OH)_2_D_3_. We therefore knocked-down AMPK⍺1 levels in MCF-7 cells and investigated TXNIP protein expression in response to a 24- and 72-h treatment with 1,25(OH)_2_D_3_. Interestingly, the reduction in TXNIP expression by 1,25(OH)_2_D_3_ was observed in cells with knocked-down AMPK⍺1 levels (Fig. 5f).

Additionally, we postulated that TXNIP degradation by 1,25(OH)_2_D_3_ may be Ca^2+^-dependent, for example, by increasing intracellular Ca^2+^ levels leading to AMPK activation or by activating Ca^2+^-dependent proteases, namely calpains. To this end, we employed calcipotriol, a hypo-calcemic analog of 1,25(OH)_2_D_3_, as well as the cell permeable Ca^2+^ chelator BAPTA-AM. Similar to the parental molecule, calcipotriol treatment led to a reduction in TXNIP levels in MCF-7 cells (Fig. 5g). Moreover, addition of BAPTA-AM to the conditioned medium of 1,25(OH)_2_D_3_-treated MCF-7 cells did not rescue TXNIP levels (Fig. 5g). These data indicate the lack of involvement of Ca^2+^ signaling in mediating 1,25(OH)_2_D_3_’s effects on TXNIP expression.

### Negative regulation of TXNIP protein levels by 1,25(OH)_2_D_3_ is possibly ER dependent

**Fig. 6:**
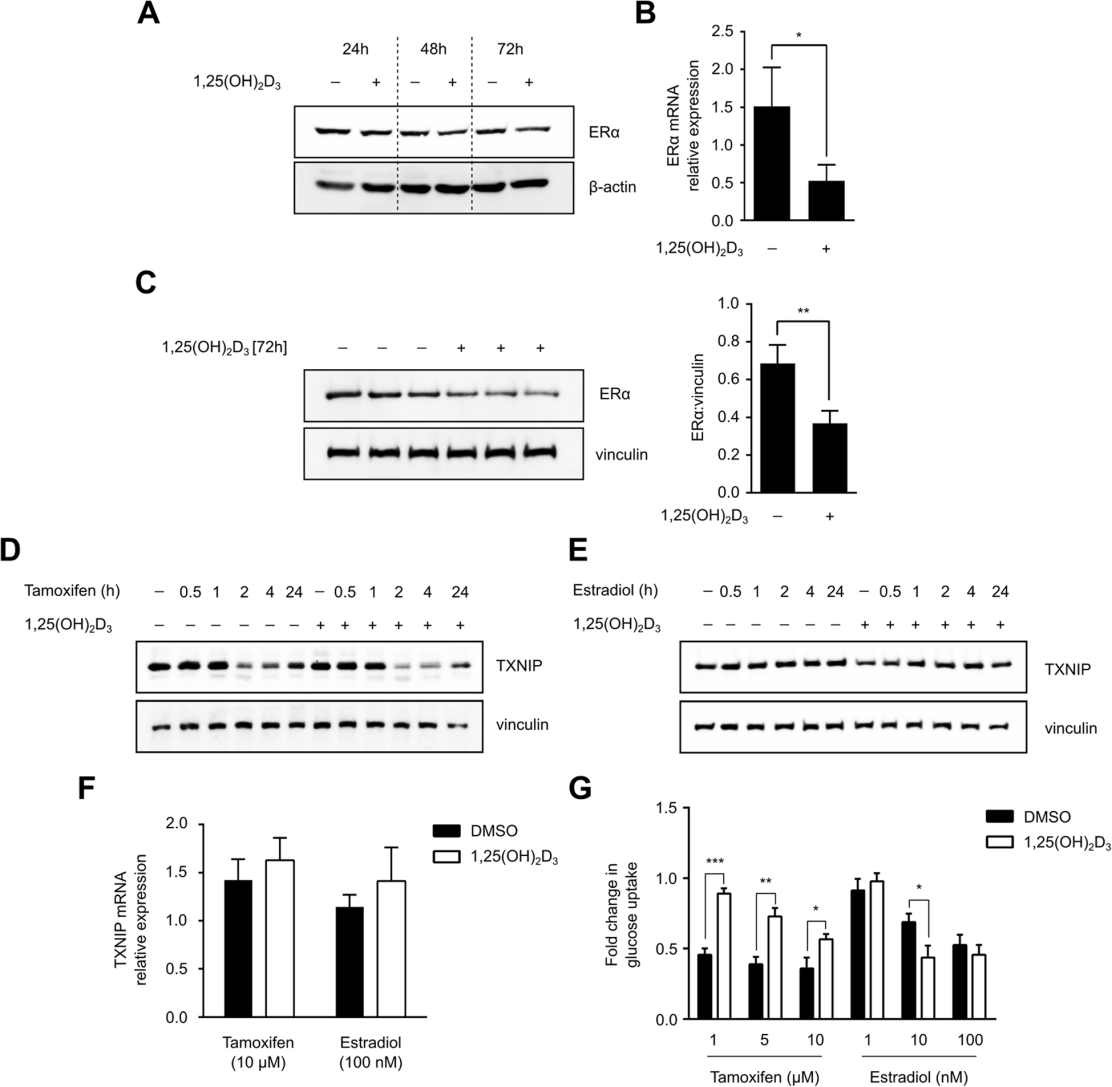
Reduction of TXNIP expression in MCF-7 cells by 1,25(OH)_2_D_3_ is possibly ER-dependent. **a**–**c** 1,25(OH)_2_D_3_ (100 nM) treatment significantly reduces ER⍺ mRNA (72 h) and protein expression in MCF-7 cells. Relative expression was calculated using the ∆∆Ct method, with vinculin as the housekeeping gene. Statistical significance between DMSO- and 1,25(OH)_2_D_3_-treated cells is calculated using a two-tailed Student’s *t* test, where *p* values less than or equal to 0.05, 0.01, and 0.001 are depicted in the figures by *, **, and ***, respectively. Error bars ± SD; *n* = 3. Tamoxifen (10 μM) treatment (**d**), but not estradiol (100 nM) (**e**), reduces TXNIP protein expression in a time-dependent manner. **f** Non-significant regulation of TXNIP mRNA levels is observed in MCF-7 cells treated for 24 h with either tamoxifen or estradiol, alone and in combination with 1,25(OH)_2_D_3_. **g** Glucose uptake in MCF-7 cells is significantly reduced by various concentrations of tamoxifen (24 h), an effect that is significantly ablated in the presence of 1,25(OH)_2_D_3_. Estradiol reduces glucose uptake in a concentration-dependent manner, with 1,25(OH)_2_D_3_ only influencing regulation of glucose uptake in response to 10 nM treatment with estradiol

Previous reports have highlighted the ability of 1,25(OH)_2_D_3_ to decrease the expression of ER⍺ in BCa cells [47, 48]. Since estradiol stimulates glucose uptake and metabolism in BCa cells [49, 50], we investigated the possible involvement of ER in 1,25(OH)_2_D_3_’s regulation of TXNIP. Firstly, we sought to confirm the reduction in ER⍺ mRNA and protein expression in MCF-7 cells by 1,25(OH)_2_D_3_. Indeed, treatment was found to reduce ER⍺ expression in MCF-7 cells (Fig. 6a–c). We then co-treated cells with ER signaling regulators, namely the selective ER modulator tamoxifen, and estradiol, in the presence of either DMSO or 1,25(OH)_2_D_3_. Tamoxifen, independent of 1,25(OH)_2_D_3_, was found to reduce TXNIP protein levels in a time-dependent manner (Fig. 6d). Estradiol on the other hand did not markedly influence TXNIP protein levels in either DMSO- or 1,25(OH)_2_D_3_-treated cells (Fig. 6e). On the mRNA level, both ER agonist and antagonist did not significantly alter TXNIP expression, independent of 1,25(OH)_2_D_3_ (Fig. 6f).

Given TXNIP’s role in glucose homeostasis, and since GLUT1 expression has been shown to be induced by estradiol treatment [51], we aimed to study the influence of co-treating MCF-7 cells with ER modulators and either DMSO or 1,25(OH)_2_D_3_ on glucose uptake. While different concentrations of tamoxifen markedly reduced glucose uptake, 1,25(OH)_2_D_3_ co-treatment was found to significantly attenuate this effect (Fig. 6g). Estradiol treatment on the other hand led to a concentration-dependent reduction in glucose uptake, an effect that was not largely impacted by 1,25(OH)_2_D_3_ co-treatment (Fig. 6g).

## Discussion

Calcitriol and its analogues are known to strongly influence the survival of BCa cells through multiple mechanisms [3, 4]. Recent studies have illustrated the ability of this molecule to influence energy utilization of different cancerous and non-cancerous experimental models, including transformed breast epithelial cells [13–16, 52, 53]. In the current study, we report 1,25(OH)_2_D_3_-induced metabolic reprogramming of BCa cell lines representing distinct molecular subtypes. We performed in-depth analyses of 1,25(OH)_2_D_3_’s metabolic effects using a combination of biosensor technology, GC/MS-based metabolomics, gene expression analysis, and assessment of overall energy levels. We show that although metabolic rewiring occurs in all investigated cell lines in response to treatment, both similarities and differences in the metabolic phenotypes are observed. For example, while differential regulation of glucose and amino acid metabolism, as well as energy-related signaling molecules was observed, 1,25(OH)_2_D_3_ was found to induce G6PD expression and activity in all cell lines.

Findings of the current study demonstrate that modulation of metabolic networks may be a mechanism through which 1,25(OH)_2_D_3_ exerts anti-tumor effects. Importantly, results highlight certain metabolic “weak points” that 1,25(OH)_2_D_3_-treated cells exhibit, which could be candidate targets for combination chemotherapy. We propose that exploiting such metabolic alterations may be key to improving the outcome of vitamin D-based therapies in clinical trials of cancer treatment. For example, induction in the expression and activity of the metabolic oncogene G6PD by 1,25(OH)_2_D_3_ may enhance cancer cells’ survival, due to the generated NADPH, which could be used for reductive biosynthesis and anti-oxidant defense [26]. NADPH may also contribute to xenobiotic metabolism and elimination by cytochrome P450 enzymes [26] and thus may also amplify CYP24A1-dependent degradation of 1,25(OH)_2_D_3_. Therefore, G6PD-inhibiting therapeutic regimens may hamper 1,25(OH)_2_D_3_’s catabolism on the one hand, as well as limit cellular growth on the other. Noteworthy is that while 1,25(OH)_2_D_3_ (72 h) did not substantially inhibit MCF-7 proliferation (Fig. 2b)—supporting a counter-therapeutic role for G6PD activation by treatment—intracellular ROS levels were found to be induced by 1,25(OH)_2_D_3_ (Fig. 3d–g), indicating that the increasing NADPH production may not have been used for anti-oxidant defense. Studies have illustrated a context-dependent role of 1,25(OH)_2_D_3_ in regulating redox balance, with both pro- and anti-oxidant effects reported in different experimental systems [28, 42, 54]. Furthermore, while NADPH has been mainly associated with ROS detoxification, it may also be used by NADPH oxidases to produce ROS [55]. Importantly, 1,25(OH)_2_D_3_ was not found to hamper the reduction in cellular proliferation by genetic inhibition of G6PD and was found to enhance the anti-proliferative effects of DHEA.

Another target for combinatorial strategies in vitamin D-based chemotherapies is serine metabolism. Combination of 1,25(OH)_2_D_3_ with either 5-FU or CBR-5884 was found to be more potent than the corresponding mono-treatments (Fig. 2e), despite 1,25(OH)_2_D_3_ alone leading to serine accumulation (Fig. 1b), and upregulation of expression of serine metabolism-related genes in MCF-7 cells (Fig. 2d). Although aberrations in serine metabolism have been previously linked to chemo-resistance [56, 57], we postulate that the demonstrated diversity of 1,25(OH)_2_D_3_’s metabolic effects may enable anti-cancer drugs overcome this potential therapeutic hurdle.

We also investigated the regulation of TXNIP by 1,25(OH)_2_D_3_ in BCa cells. Although initially described as the VDUP1 [35], it remained unclear if 1,25(OH)_2_D_3_ induces TXNIP expression in different cell types since this canonical regulation has only been observed in HL-60 cells [58], and promoter analysis of mouse VDUP1 did not demonstrate the presence of VDRE [59]. Additionally, we have recently shown that calcitriol induces non-canonical regulation of TXNIP in the prostate cancer cell line LNCaP [16], and that in HL-60 cells, i.e., the cell line in which the original identification was made, induction in TXNIP protein levels by 1,25(OH)_2_D_3_ depended on the availability of glucose in the culture medium [40]. Furthermore, screening TXNIP regulation by 1,25(OH)_2_D_3_ in multiple cancer cell lines of different tissue origins demonstrated either induction, reduction, or not change in TXNIP levels by treatment [40]. In the present study, we observed that not all BCa cell lines exhibit detectable levels of TXNIP, and in those that do, treatment either reduces or has no clear effect on TXNIP protein expression (Fig. 3a). These findings indicate that TXNIP, a putative tumor suppressor, may not be central to calcitriol’s anti-tumor effects. Moreover, the reduction in TXNIP levels by 1,25(OH)_2_D_3_ in MCF-7 contrasts the accumulation of intracellular ROS since TXNIP is known to bind to reduced thioredoxin, thus inhibiting its anti-oxidant activity.

## Conclusions

In spite of many studies demonstrating the ability of calcitriol to inhibit BCa cell survival, few have focused on the molecule’s role in regulating cells’ energy utilization and nutrient-sensing. 1,25(OH)_2_D_3_ appears to induce diverse changes in the levels and/or activity of key enzymes, metabolites, and signaling molecules involved in energy metabolism, leading to multi-faceted metabolic phenotypes in BCa cells. Distinct metabolic vulnerabilities have been identified in 1,25(OH)_2_D_3_-treated BCa cells, which may serve as targets for combination chemotherapy. For example, by combining 1,25(OH)_2_D_3_ with G6PD-inhibiting regimens, substantial anti-tumor effects were observed. Additionally, while TXNIP has been previously identified as a tumor suppressor and a vitamin D target gene, our results indicate that activation of TXNIP expression by calcitriol is not achieved in different BCa cell lines, and that in MCF-7 cells, the reduction in the TXNIP level by treatment is potentially due to rewiring of glucose metabolism, protein degradation, and cross-talk between VDR and ER signaling.

## Additional files

Additional file 1: **Table S1.** List of primers used for mRNA expression analysis. (DOCX 18 kb)

Additional file 2: **Table S2.** List of siRNA sequences used in the study. (DOCX 13 kb)

Additional file 3: **Movie S1.** Showing the development of MitoSOX red fluorescence in DMSO-treated MCF-7 cells. (AVI 9250 kb)

Additional file 4: **Movie S2.** Showing the development of MitoSOX red fluorescence in 1,25(OH)_2_D_3_-treated MCF-7 cells. (AVI 7982 kb)

Additional file 5: **Figure S1.** 1,25(OH)_2_D_3_ (100 nM) significantly reduces glucose uptake in MCF-7 cells after 24, but not 72 h of treatment. Statistical significance between DMSO- and 1,25(OH)_2_D_3_-treated cells is calculated using a two-tailed Student’s *t* test, where *p* values less than or equal to 0.01 are depicted in the figure by **. Error bars ± SD; *n* = 3. (TIFF 436 kb)

## Abbreviations

1,25(OH)_2_D_3_: 1,25-Dihydroxyvitamin D_3_
2-NBDG: 2-[N-(7-nitrobenz-2-oxa-1,3-diazol-4-yl) amino]-2-deoxy-D-glucose
3PG: 3-Phosphoglycerate
3PHP: 3-Phosphopyruvate
3PS: 3-Phosphoserine
5-FU: 5-Fluorouracil
AMPK: AMP-activated protein kinase
BCa: Breast cancer
DHE: Dihydroethidium
DHEA: Dehydroepiandrosterone
ER: Estrogen receptor
G6PD: Glucose-6-phosohate dehydrogenase
MCT: Monocarboxylate transporter
PEP: Phosphoenolpyruvate
PHGDH: Phosphoglycerate dehydrogenase
PPP: Pentose phosphate pathway
PSAT1: Phosphoserine aminotransferase 1
PSPH: Phosphoserine phosphatase
RM: Running medium
ROS: Reactive oxygen species
SHMT1/2: Serine hydroxymethyltransferase 1/2
SSP: Serine synthesis pathway
TNBC: Triple-negative breast cancer
TXNIP: Thioredoxin-interacting protein
TYMS: Thymidylate synthase
VDR: Vitamin D receptor
VDRE: Vitamin D response elements
VDUP1: Vitamin D_3_-upregulated protein 1

## References

[1] Torre LA, Bray F, Siegel RL, Ferlay J, Lortet-Tieulent J, Jemal A (2015). Global cancer statistics, 2012. CA Cancer J Clin.

[2] Howell A, Anderson AS, Clarke RB, Duffy SW, Evans DG, Garcia-Closas M, Gescher AJ, Key TJ, Saxton JM, Harvie MN (2014). Risk determination and prevention of breast cancer. Breast Cancer Res.

[3] Krishnan AV, Swami S, Feldman D (2010). Vitamin D and breast cancer: inhibition of estrogen synthesis and signaling. J Steroid Biochem Mol Biol.

[4] Welsh J. Vitamin D and breast cancer: past and present. J Steroid Biochem Mol Biol. 2018;177:15–20. 10.1016/j.jsbmb.2017.07.025 PMC5780261 28746837

[5] Holick MF (2007). Vitamin D deficiency. N Engl J Med.

[6] Feldman D, Krishnan AV, Swami S, Giovannucci E, Feldman BJ (2014). The role of vitamin D in reducing cancer risk and progression. Nat Rev Cancer.

[7] Perou CM, Sorlie T, Eisen MB, van de Rijn M, Jeffrey SS, Rees CA, Pollack JR, Ross DT, Johnsen H, Akslen LA, Fluge O, Pergamenschikov A, Williams C, Zhu SX, Lonning PE, Borresen-Dale AL, Brown PO, Botstein D (2000). Molecular portraits of human breast tumours. Nature.

[8] Cancer Genome Atlas N (2012). Comprehensive molecular portraits of human breast tumours. Nature.

[9] Toss A, Cristofanilli M (2015). Molecular characterization and targeted therapeutic approaches in breast cancer. Breast Cancer Res.

[10] Lloyd SM, Arnold J, Sreekumar A (2015). Metabolomic profiling of hormone-dependent cancers: a bird’s eye view. Trends Endocrinol Metab.

[11] Mishra P, Ambs S. Metabolic signatures of human breast cancer. Mol Cell Oncol. 2015;2:e992217. 10.4161/23723556.2014.992217 PMC4438683 26005711

[12] Morandi A, Indraccolo S (1868). Linking metabolic reprogramming to therapy resistance in cancer. Biochim Biophys Acta.

[13] Wilmanski T, Buhman K, Donkin SS, Burgess JR, Teegarden D (2017). 1alpha,25-dihydroxyvitamin D inhibits de novo fatty acid synthesis and lipid accumulation in metastatic breast cancer cells through down-regulation of pyruvate carboxylase. J Nutr Biochem.

[14] Zheng W, Tayyari F, Gowda GA, Raftery D, McLamore ES, Shi J, Porterfield DM, Donkin SS, Bequette B, Teegarden D (2013). 1,25-dihydroxyvitamin D regulation of glucose metabolism in Harvey-ras transformed MCF10A human breast epithelial cells. J Steroid Biochem Mol Biol.

[15] Zhou X, Zheng W, Nagana Gowda GA, Raftery D, Donkin SS, Bequette B, Teegarden D (2016). 1,25-Dihydroxyvitamin D inhibits glutamine metabolism in Harvey-ras transformed MCF10A human breast epithelial cell. J Steroid Biochem Mol Biol.

[16] Abu El Maaty MA, Alborzinia H, Khan SJ, Buttner M, Wolfl S. 1,25(OH)2D3 Disrupts glucose metabolism in prostate cancer cells leading to a truncation of the tca cycle and inhibition of txnip expression. Biochim Biophys Acta. 2017;1864:1618-30. 10.1016/j.bbamcr.2017.06.019 28651973

[17] Abu El Maaty MA, Wolfl S. Vitamin D as a novel regulator of tumor metabolism: insights on potential mechanisms and implications for anti-cancer therapy. Int J Mol Sci. 2017;18:2184. 10.3390/ijms18102184 PMC5666865 29048387

[18] Alborzinia H, Can S, Holenya P, Scholl C, Lederer E, Kitanovic I, Wolfl S (2011). Real-time monitoring of cisplatin-induced cell death. PLoS One.

[19] Jiang P, Du W, Wang X, Mancuso A, Gao X, Wu M, Yang X (2011). p53 regulates biosynthesis through direct inactivation of glucose-6-phosphate dehydrogenase. Nat Cell Biol.

[20] Tian WN, Braunstein LD, Pang J, Stuhlmeier KM, Xi QC, Tian X, Stanton RC (1998). Importance of glucose-6-phosphate dehydrogenase activity for cell growth. J Biol Chem.

[21] Abu El Maaty MA, Strassburger W, Qaiser T, Dabiri Y, Wolfl S. Differences in P53 status significantly influence the cellular response and cell survival to 1,25-dihydroxyvitamin D3-metformin cotreatment in colorectal cancer cells. Mol Carcinog. 2017;56:​2486-98. 10.1002/mc.22696 28618116

[22] Cheng X, Merz KH, Vatter S, Zeller J, Muehlbeyer S, Thommet A, Christ J, Wolfl S, Eisenbrand G (2017). Identification of a water-soluble indirubin derivative as potent inhibitor of insulin-like growth factor 1 receptor through structural modification of the parent natural molecule. J Med Chem.

[23] Gyorffy B, Lanczky A, Eklund AC, Denkert C, Budczies J, Li Q, Szallasi Z (2010). An online survival analysis tool to rapidly assess the effect of 22,277 genes on breast cancer prognosis using microarray data of 1,809 patients. Breast Cancer Res Treat.

[24] Berkers CR, Maddocks OD, Cheung EC, Mor I, Vousden KH (2013). Metabolic regulation by p53 family members. Cell Metab.

[25] Maiuri MC, Galluzzi L, Morselli E, Kepp O, Malik SA, Kroemer G (2010). Autophagy regulation by p53. Curr Opin Cell Biol.

[26] Stanton RC (2012). Glucose-6-phosphate dehydrogenase, NADPH, and cell survival. IUBMB Life.

[27] Patra KC, Hay N (2014). The pentose phosphate pathway and cancer. Trends Biochem Sci.

[28] Bao BY, Ting HJ, Hsu JW, Lee YF (2008). Protective role of 1 alpha, 25-dihydroxyvitamin D3 against oxidative stress in nonmalignant human prostate epithelial cells. Int J Cancer.

[29] Noun A, Garabedian M, Monet JD (1989). Stimulatory effect of 1,25-dihydroxyvitamin D3 on the glucose-6-phosphate dehydrogenase activity in the MCF-7 human breast cancer cell line. Cell Biochem Funct.

[30] Simmons KM, Beaudin SG, Narvaez CJ, Welsh J (2015). Gene signatures of 1,25-dihydroxyvitamin D3 exposure in normal and transformed mammary cells. J Cell Biochem.

[31] Yang M, Vousden KH (2016). Serine and one-carbon metabolism in cancer. Nat Rev Cancer.

[32] Fang Z, Jiang C, Feng Y, Chen R, Lin X, Zhang Z, Han L, Chen X, Li H, Guo Y, Jiang W (2016). Effects of G6PD activity inhibition on the viability, ROS generation and mechanical properties of cervical cancer cells. Biochim Biophys Acta.

[33] Vander Heiden MG (2011). Targeting cancer metabolism: a therapeutic window opens. Nat Rev Drug Discov.

[34] Mullarky E, Lucki NC, Beheshti Zavareh R, Anglin JL, Gomes AP, Nicolay BN, Wong JC, Christen S, Takahashi H, Singh PK, Blenis J, Warren JD, Fendt SM, Asara JM, DeNicola GM, Lyssiotis CA, Lairson LL, Cantley LC (2016). Identification of a small molecule inhibitor of 3-phosphoglycerate dehydrogenase to target serine biosynthesis in cancers. Proc Natl Acad Sci U S A.

[35] Chen KS, DeLuca HF (1994). Isolation and characterization of a novel cDNA from HL-60 cells treated with 1,25-dihydroxyvitamin D-3. Biochim Biophys Acta.

[36] Zhou J, Yu Q, Chng WJ (2011). TXNIP (VDUP-1, TBP-2): a major redox regulator commonly suppressed in cancer by epigenetic mechanisms. Int J Biochem Cell Biol.

[37] Stoltzman CA, Peterson CW, Breen KT, Muoio DM, Billin AN, Ayer DE (2008). Glucose sensing by MondoA:Mlx complexes: a role for hexokinases and direct regulation of thioredoxin-interacting protein expression. Proc Natl Acad Sci U S A.

[38] Wu N, Zheng B, Shaywitz A, Dagon Y, Tower C, Bellinger G, Shen CH, Wen J, Asara J, McGraw TE, Kahn BB, Cantley LC (2013). AMPK-dependent degradation of TXNIP upon energy stress leads to enhanced glucose uptake via GLUT1. Mol Cell.

[39] Hardie DG, Ross FA, Hawley SA (2012). AMP-activated protein kinase: a target for drugs both ancient and modern. Chem Biol.

[40] Abu El Maaty MA, Almouhanna F, Wolfl S. Expression of TXNIP in cancer cells and regulation by 1,25(OH)(2)D(3): is it really the vitamin D(3) upregulated protein? Int J Mol Sci. 2018;19:796. 10.3390/ijms19030796 PMC5877657 29534438

[41] Zhou J, Chng WJ (2013). Roles of thioredoxin binding protein (TXNIP) in oxidative stress, apoptosis and cancer. Mitochondrion.

[42] Koren R, Hadari-Naor I, Zuck E, Rotem C, Liberman UA, Ravid A (2001). Vitamin D is a prooxidant in breast cancer cells. Cancer Res.

[43] Yu FX, Chai TF, He H, Hagen T, Luo Y (2010). Thioredoxin-interacting protein (Txnip) gene expression: sensing oxidative phosphorylation status and glycolytic rate. J Biol Chem.

[44] Schwartz AG, Pashko LL (2004). Dehydroepiandrosterone, glucose-6-phosphate dehydrogenase, and longevity. Ageing Res Rev.

[45] Alvarez-Diaz S, Larriba MJ, Lopez-Otin C, Munoz A (2010). Vitamin D: proteases, protease inhibitors and cancer. Cell Cycle.

[46] Zhang P, Wang C, Gao K, Wang D, Mao J, An J, Xu C, Wu D, Yu H, Liu JO, Yu L (2010). The ubiquitin ligase itch regulates apoptosis by targeting thioredoxin-interacting protein for ubiquitin-dependent degradation. J Biol Chem.

[47] Swami S, Krishnan AV, Feldman D (2000). 1alpha,25-dihydroxyvitamin D3 down-regulates estrogen receptor abundance and suppresses estrogen actions in MCF-7 human breast cancer cells. Clin Cancer Res.

[48] Swami S, Krishnan AV, Peng L, Lundqvist J, Feldman D (2013). Transrepression of the estrogen receptor promoter by calcitriol in human breast cancer cells via two negative vitamin D response elements. Endocr Relat Cancer.

[49] Imbert-Fernandez Y, Clem BF, O'Neal J, Kerr DA, Spaulding R, Lanceta L, Clem AL, Telang S, Chesney J (2014). Estradiol stimulates glucose metabolism via 6-phosphofructo-2-kinase (PFKFB3). J Biol Chem.

[50] Ko BH, Paik JY, Jung KH, Lee KH (2010). 17beta-estradiol augments 18F-FDG uptake and glycolysis of T47D breast cancer cells via membrane-initiated rapid PI3K-Akt activation. J Nucl Med.

[51] Rivenzon-Segal D, Boldin-Adamsky S, Seger D, Seger R, Degani H (2003). Glycolysis and glucose transporter 1 as markers of response to hormonal therapy in breast cancer. Int J Cancer.

[52] Ferreira GB, Vanherwegen AS, Eelen G, Gutierrez AC, Van Lommel L, Marchal K, Verlinden L, Verstuyf A, Nogueira T, Georgiadou M, Schuit F, Eizirik DL, Gysemans C, Carmeliet P, Overbergh L, Mathieu C. Vitamin D3 induces tolerance in human dendritic cells by activation of intracellular metabolic pathways. Cell Rep. 2015;10:711–25. 10.1016/j.celrep.2015.01.013 25660022

[53] Ryan ZC, Craig TA, Folmes CD, Wang X, Lanza IR, Schaible NS, Salisbury JL, Nair KS, Terzic A, Sieck GC, Kumar R (2016). 1alpha,25-dihydroxyvitamin D3 regulates mitochondrial oxygen consumption and dynamics in human skeletal muscle cells. J Biol Chem.

[54] Jeon SM, Shin EA (2018). Exploring vitamin D metabolism and function in cancer. Exp Mol Med.

[55] Reczek CR, Chandel NS (2017). The two faces of reactive oxygen species in cancer. Annu Rev Cancer Biol.

[56] Zaal EA, Wu W, Jansen G, Zweegman S, Cloos J, Berkers CR (2017). Bortezomib resistance in multiple myeloma is associated with increased serine synthesis. Cancer Metab.

[57] Vie N, Copois V, Bascoul-Mollevi C, Denis V, Bec N, Robert B, Fraslon C, Conseiller E, Molina F, Larroque C, Martineau P, Del Rio M, Gongora C (2008). Overexpression of phosphoserine aminotransferase PSAT1 stimulates cell growth and increases chemoresistance of colon cancer cells. Mol Cancer.

[58] Shalev A (2014). Minireview: thioredoxin-interacting protein: regulation and function in the pancreatic beta-cell. Mol Endocrinol.

[59] Ludwig DL, Kotanides H, Le T, Chavkin D, Bohlen P, Witte L (2001). Cloning, genetic characterization, and chromosomal mapping of the mouse VDUP1 gene. Gene.

