# Supplementary material for: Activation of pro-survival metabolic networks by 1,25(OH)2D3 does not hamper the sensitivity of breast cancer cells to chemotherapeutics
Source: Cancer Metab. 2018 Aug 30;6:11. doi: 10.1186/s40170-018-0183-6 (PMC6116450; doi:10.1186/s40170-018-0183-6)
Supplement: Supplementary file 1 — Table S1. List of primers used for mRNA expression analysis. (DOCX 18 kb) [file 40170_2018_183_MOESM1_ESM.docx]

**Table S1** List of primers used for mRNA expression analysis.

| Primer name | Primer sequence |
| --- | --- |
| **ACACA-For** | 5'-GATTCGTTGTCATGGTCACACC-3' |
| **ACACA-Rev** | 5'-GTTGTTGTTTGGTCCTCCAGG-3' |
| **ACLY-For** | 5'-GCATCTATGCCACCCGAGAAG-3' |
| **ACLY-Rev** | 5'-CCAACAAGCAGCTTCTGGGC-3' |
| **AMPKb1-For** | 5'-GTCGCTGAGGGGTGGTGAAG-3' |
| **AMPKb1-Rev** | 5'-CTCGCAATCGCGCTTTACCTG-3' |
| **BAD-For** | 5'-CGGAGGATGAGTGACGAGTT-3' |
| **BAD-Rev** | 5'-CCAAGTTCCGATCCCACCAG-3' |
| **BAX-For** | 5'-GGGGACGAACTGGACAGTAA-3' |
| **BAX-Rev** | 5'-CAGTTGAAGTTGCCGTCAGA-3' |
| **BECN1-For** | 5'-GCTGAAGACAGAGCGATGGTA-3' |
| **BECN1-Rev** | 5'-GGACGTCTTAGACCCTTCCA-3' |
| **COX5B-For** | 5'-CCAGCGTCGTCTGGTTTTGG-3' |
| **COX5B-Rev** | 5'-GCTCAGTGTGCCAGCTGCT-3' |
| **CYP24A1-For** | 5'-TGGGGCTGGGAGTAATACTGA-3' |
| **CYP24A1-Rev** | 5'-GAACGCAATTTCATGGGAGGC-3' |
| **ESR1-For** | 5'-CGCTGCGTCGCCTCTAA-3' |
| **ESR1-Rev** | 5'-TTGGTGTGGAGGGTCATGGT-3' |
| **G6PD-For** | 5'-CCAAGAACATTCACGAGTCCTG-3' |
| **G6PD-Rev** | 5'-GGACAGCCGGTCAGAGCTC-3' |
| **GLS1-For** | 5'-CCTCAACTGGCCAAATTCAGTC-3' |
| **GLS1-Rev** | 5'-CTGAAGACAGAAGGGAACTTTG-3' |
| **GLS2-For** | 5'-GCTTTCCTAAGGGGGTGGAC-3' |
| **GLS2-Rev** | 5'-GTGGCTGCCATGACACTGC-3' |
| **GLUT1-For** | 5'-CAGTTTGGCTACAACACTGGAG-3' |
| **GLUT1-Rev** | 5'-GCAGGATGCTCTCCCCATAG-3' |
| **GOT1-For** | 5'-CACGAGTATCTGCCAATCCTG-3' |
| **GOT1-Rev** | 5'-CCTACCCGCTTCTCCTTGAG-3' |
| **HKII-For** | 5'-GTGGCACCCAGCTGTTTGAC-3' |
| **HKII-Rev** | 5'-CGAGAAGGTAAAACCCAGTGG-3' |
| **ITCH-For** | 5'-5TCTAGTAGCTGTGGTCGGGG-3' |
| **ITCH-Rev** | 5'-CACAAGGCCACCGTGAAATG-3' |
| **LDHA-For** | 5'-GAAGGGAGAGATGATGGATCTC-3' |
| **LDHA-Rev** | 5'-CTTATCTTCCAAGCCACGTAGG-3' |
| **MYC-For** | 5'-TTCGGGTAGTGGAAAACCAG-3' |
| **MYC-Rev** | 5'-CAGCAGCTCGAATTTCTTCC-3' |
| **NDUFA1-For** | 5'-GAGATTCTCCCCGGACTCTC-3' |
| **NDUFA1-Rev** | 5'-CCCTTTTTTCCTTGCCCCCG-3' |
| **OGDH-For** | 5'-GGTAGAAGCACAGCCCAACG-3' |
| **OGDH-Rev** | 5'-CTGTGCTACATGGTGCCCTC-3' |
| **P21-For** | 5'-GACACCACTGGAGGGTGACT-3' |
| **P21-Rev** | 5'-CAGGTCCACATGGTCTTCCT-3' |
| **PDHA1-For** | 5'-CCCCACAGACCATCTCATCAC-3' |
| **PDHA1-Rev** | 5'-CCTTTTCGTCCTGTAAGCTCTG-3' |
| **PDHK1-For** | 5'-GGATCGATTCTACATGAGTCGC-3' |
| **PDHK1-Rev** | 5'-GTTTTCGATGAGATGGACTTCC-3' |
| **PHGDH-For** | 5'-CCGTCCAATCAAAAGGAGACTG-3' |
| **PHGDH-Rev** | 5'-GAATCGGCCGCTGTGAGTAG-3' |
| **PKM2-For** | 5'-CAGAGGCTGCCATCTACCAC-3' |
| **PKM2-Rev** | 5'-GAGGACGATTATGGCCCCAC-3' |
| **PSAT1-For** | 5'-GTCCAGTGGAGCCCCAAAAT-3' |
| **PSAT1-Rev** | 5'-TCCCACAGACCTATGCCCTT-3' |
| **PSPH-For** | 5'-TGAGACGTAAGAACCTGCCC-3' |
| **PSPH-Rev** | 5'-TCTTGAGATCTGCTTAAAAGGGAGT-3' |
| **PUMA-For** | 5'-GACGACCTCAACGCACAGTA-3' |
| **PUMA-Rev** | 5'-CACCTAATTGGGCTCCATCT-3' |
| **SDHC-For** | 5'-GGACAACCACTCCAGACTGG-3' |
| **SDHC-Rev** | 5'-GGACAACCACTCCAGACTGG-3' |
| **SHMT1-For** | 5'-CAAGTTCGGGGTTTGGGGTT-3' |
| **SHMT1-Rev** | 5'-TCGAAGCTGCCTAGCGAC-3' |
| **SHMT2-For** | 5'-ATCCCTCCCGTTAGCTTTGG-3' |
| **SHMT2-Rev** | 5'-TACAGCATCGCAACTCGGAA-3' |
| **SLC1A4-For** | 5'-TCCTCGCCTTTCTCGCAC-3' |
| **SLC1A4-Rev** | 5'-AAAAGACGGGGTTCCCAATGA-3' |
| **SLC1A5-For** | 5'-CTCGAAGCAGTCAACCTCCC-3' |
| **SLC1A5-Rev** | 5'-ACTCCGTACGGTCCACGTAA-3' |
| **SREBP1-For** | 5'-CATTGAGCTCCTCTCTTGAAGC-3' |
| **SREBP1-Rev** | 5'-GGGTACATCTTCAATGGAGTGG-3' |
| **TFAM-For** | 5'-TGTCTGACTCTGAAAAGGAAGTGA-3' |
| **TFAM-Rev** | 5'-ACATGAATCAAGCCTTGCCC-3' |
| **TIGAR-For** | 5'-AAGTGGTGTGGGGGAGGTAG-3' |
| **TIGAR-Rev** | 5'-CGGACAACAGTCAGAGCGAA-3' |
| **TSC2-For** | 5'-CGTAGGCTACCCCGAGCA-3' |
| **TSC2-Rev** | 5'-CAGACAGATTTCTCGTCCGC-3' |
| **TXNIP-For** | 5'-CGCCTCCTGCTTGAAACTAAC-3' |
| **TXNIP-Rev** | 5'-AATATACGCCGCTGGTTACACT-3' |
| **TYMS-For** | 5'-GCACCCTGTCGGTATTCGG-3' |
| **TYMS-Rev** | 5'-GCAACTCCTCCAAAACACCC-3' |
| **VCL-For** | 5'-CAGTCAGACCCTTACTCAGTG-3' |
| **VCL-Rev** | 5'-CAGCCTCATCGAAGGTAAGGA-3' |
| **VDR-For** | 5'-TGGAGACTTTGACCGGAACG-3' |
| **VDR-Rev** | 5'-GGGCAGGTGAATAGTGCCTT-3' |
