# Supplementary material for: Activation of pro-survival metabolic networks by 1,25(OH)2D3 does not hamper the sensitivity of breast cancer cells to chemotherapeutics
Source: Cancer Metab. 2018 Aug 30;6:11. doi: 10.1186/s40170-018-0183-6 (PMC6116450; doi:10.1186/s40170-018-0183-6)
Supplement: Supplementary file 2 — Table S2. List of siRNA sequences used in the study. (DOCX 13 kb) [file 40170_2018_183_MOESM2_ESM.docx]

**Table S2** List of siRNA sequences used in the study.

| TARGET | SENSE (5'-3') | ANTISENSE (5'-3') |
| --- | --- | --- |
| **AMPKα1** | GGGGGCCACAAUCAAAGAUAU  GGGGGAUUGGAACAUGAUUUA  GGGGGAGAGCUAUUUGAUUAUA | AUAUCUUUGAUUGUGGCCCCC  UAAAUCAUGUUCCAAUCCCCC  UAUAAUCAAAUAGCUCUCCCCC |
| **G6PD** | GGGGGCCAUUAAAUCCGCAAACA  GGGGGCAGUCGGAUACACACAUAU  GGGGGCAACAGAUACAAGAACGU | UGUUUGCGGAUUUAAUGGCCCCC  AUAUGUGUGUAUCCGACUGCCCCC  ACGUUCUUGUAUCUGUUGCCCCC |
