# Supplementary figures and images for: Activation of pro-survival metabolic networks by 1,25(OH)2D3 does not hamper the sensitivity of breast cancer cells to chemotherapeutics
Source: Cancer Metab. 2018 Aug 30;6:11. doi: 10.1186/s40170-018-0183-6 (PMC6116450; doi:10.1186/s40170-018-0183-6)

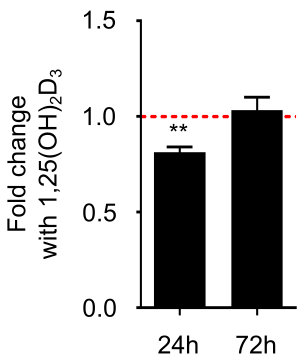

Supplement: Supplementary file 5 — Figure S1. 1,25(OH)2D3 (100 nM) significantly reduces glucose uptake in MCF-7 cells after 24, but not 72 h of treatment. Statistical significance between DMSO- and 1,25(OH)2D3-treated cells is calculated using a two-tailed Student’s t test, where p values less than or equal to 0.01 are depicted in the figure by **. Error bars ± SD; n = 3. (TIFF 436 kb) [file 40170_2018_183_MOESM5_ESM.tiff]
